# Adsorption and precipitation of ODA on KCl surfaces during flotation: evidence from FTIR and XPS

**DOI:** 10.1039/d6ra08381a

**Published:** 2026-11-02

**Authors:** Huaide Cheng, Changhao Luo, Qingyu Hai

**Affiliations:** a Key Laboratory of Green and High-end Utilization of Salt Lake Resources, Qinghai Institute of Salt Lakes, Chinese Academy of Sciences No 18 Xinning Road Xining 810008 China chenghuaide@isl.ac.cn; b Qinghai Provincial Key Laboratory of Geology and Environment of Salt Lakes Xining 810008 China; c University of Chinese Academy of Sciences Beijing China

## Abstract

This study investigates the concentration-dependent grain size evolution of octadecylamine hydrochloride (ODA·HCl), a commonly used cationic collector in potassium chloride flotation. FTIR and XPS spectroscopy were employed to analyze the adsorption states and interaction mechanisms of ODA on KCl surfaces under static equilibrium adsorption and dynamic flotation conditions. The results showed that: (1) the ODA·HCl flocculation revealed a pronounced concentration-driven evolution of grain size, and become smaller and more uniform when the concentration of ODA·HCl approaches that of actual flotation (1 × 10^−2^ mol L^−1^); (2) FTIR analysis confirmed the successful adsorption of ODA·HCl onto KCl surfaces, and the adsorption under dynamic flotation conditions exhibited a non-linear dependence on concentration; (3) XPS verified that ODA·HCl attaches to the KCl surface *via* a synergistic mechanism involving electrostatic attraction, partial deprotonation, and hydrogen bonding, without disrupting the KCl crystal structure. Taking into account grain size, adsorption behavior, flotation performance, and KCl dissolution loss in the mother liquor, the optimal collector concentration is proposed to lie within 1.0 × 10^−2^ to 1.6 × 10^−2^ mol L^−1^. This range achieves a high recovery (>94%), a narrow grain size distribution, low reagent consumption, and minimal potassium salt dissolution loss, providing both a theoretical basis and practical guidance for optimizing the potash flotation process.

## Abstract 2

This study investigates the concentration-dependent grain size evolution of octadecylamine hydrochloride (ODA·HCl), a commonly used cationic collector in potassium chloride flotation.

## Graphical abstract

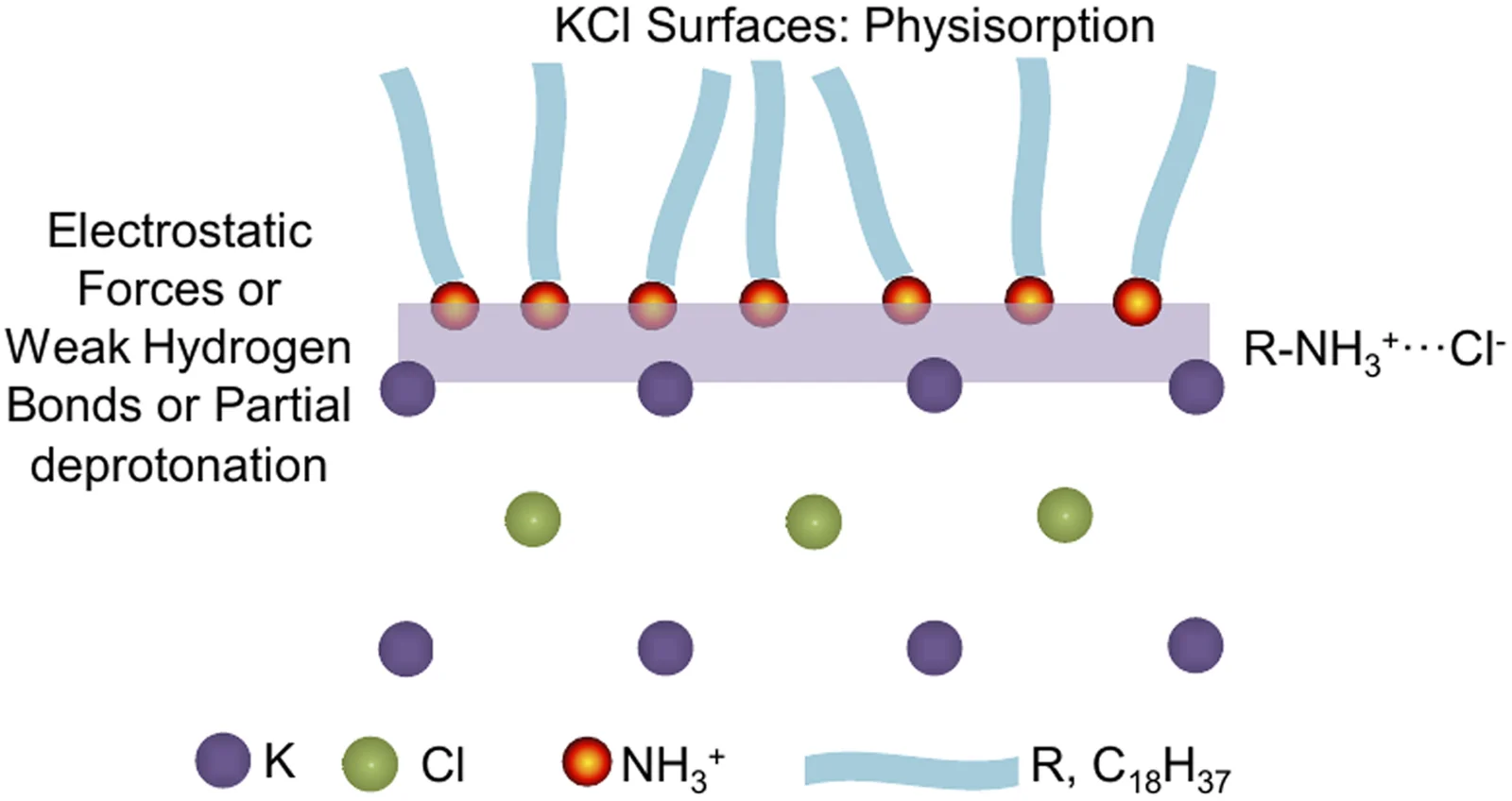

## 1 Introduction

Flotation is a complex physicochemical process involving solid, liquid, and gas phases. It exploits differences in the surface properties of minerals and uses collectors to attach the target minerals to bubbles, which act as carriers for separation.^1,2^ The key technical challenge in extracting potassium salt products (such as potassium chloride, potassium sulfate, and potassium magnesium sulfate fertilizers) from potash ores (*e.g.*, marine sedimentary deposits and salts obtained by evaporation crystallization in solar pond) lies in separating potassium salts from sodium salts.^3–6^ Common separation methods include thermal dissolution, flotation, gravity separation, and electrostatic separation, among which flotation is the most widely used.^7,8^ Globally, three-quarters of potash are produced by flotation plants.^9–12^ Although flotation can effectively enrich and recover potassium salts, considerable controversy remains regarding the surface phenomena involved in the interaction between soluble salt particles and collectors, owing to the high ionic strength of the saturated brine, the water insolubility of the collectors, the high viscosity of the brine, and the overall complexity of the brine system.

Octadecylamine (ODA), a long-chain fatty primary amine, is one of the most widely used cationic collectors in the flotation of potassium chloride.^13,14^ In industrial practice, the typical dosage is 100–120 g t^−1^, and it is usually applied as an aqueous solution with a concentration of 1 × 10^−2^ mol L^−1^ (approximately 0.3 wt%). Because of its relatively low melting point (∼50–60 °C), ODA must be preheated to 70–90 °C to melt, and then neutralized with hydrochloric acid to form the hydrochloride salt (ODA·HCl, abbreviated as ODA-H). The resulting hot emulsion or dispersion is immediately introduced into the flotation pulp at room temperature, where the selective adsorption of ODA-H at the solid–liquid interface enables effective separation of potassium salt minerals from gangue minerals.

The mechanism by which amine collectors act on salt mineral surfaces has undergone a marked shift from molecular adsorption to precipitation adhesion. Early classical theories were based primarily on ion-exchange and structure-matching models, which proposed that RNH_3_^+^ ions could occupy K^+^ lattice sites to impart hydrophobicity.^15,16^ Subsequent studies, however, revealed that the extremely low solubility of long-chain amines in saturated brines causes the collector to exist mainly as colloidal precipitates, and that flotation actually relies on the attachment of positively charged amine precipitates to negatively charged KCl surfaces through heterocoagulation.^17–19^ Research by Burdukova *et al.*^20,21^ further demonstrated that amine precipitates can also pre-adsorb at the bubble surface, thereby promoting the particle-bubble attachment process. The water-structure model^22–24^ explains from the perspective of interfacial water ordering why KCl (for structure breaking salt) can be floated whereas NaCl (for structure making salt) cannot. A more recent trend is to view adsorption and deposition as a continuous process, emphasizing the full behavioral sequence of amine collectors from the molecular state to colloidal aggregates and ultimately to surface attachment.^25,26^ The flotation separation of water-soluble salt minerals is generally conducted in saturated or supersaturated brine solutions. Understanding the flotation of salt minerals solely through differences in surface hydrophobicity is far from sufficient.^4,6,10,27–29^ Interfacial behavior in salt mineral flotation is governed by dissolution-crystallization kinetics at the salt surface, ion–ion interactions in the bulk medium, and ion hydration effects. Although significant progress has been made in interfacial water structure models, which have revealed that ion specificity is a dominant factor in brine flotation, no model currently provides a satisfactory mechanistic explanation for the adsorption process itself.^7,10,24,25,28,30–32^ Future studies should therefore more comprehensively investigate the coupling interactions among salt ions, water molecules, collectors, and salt crystals, with particular emphasis on the activity and aggregation behavior of collectors at the gas–liquid–solid interface and on clearly identifying the active sites for flotation.

Previous studies have demonstrated that ODA-based collectors are effective for KCl flotation, but they have focused mainly on practical applicability, adsorption behavior at mineral surfaces, and micellization behavior.^9,33–40^ Although these studies established the feasibility of ODA-based collectors for KCl flotation, they primarily emphasized macroscopic flotation performance, and several aspects remain insufficiently understood, including the dispersion characteristics and particle size of ODA-based collectors and the interactions between minerals and reagents. In contrast, this work systematically investigates the self-assembly aggregation behavior, particle size evolution, and the influence of the acid radical anion at different concentrations of ODA·HCl. Furthermore, using FTIR and XPS spectroscopy, the adsorption states and interaction mechanism of ODA·HCl on the potassium chloride surface are explored under static equilibrium adsorption and actual dynamic flotation conditions. We attempted to obtain new insights for building a more general flotation mechanism for salt minerals.

## 2 Experimental

### 2.1 Materials

Potassium chloride (KCl) was of ACS reagent grade (purity ≥99%) and purchased from Sigma-Aldrich. KCl was used to prepare brine solution and sieved to ≤450 µm size fraction for flotation feeding. ODA (CH_3_(CH_2_)_16_CH_2_NH_2_, 98%, Dachat Chemical Inc., Zhejiang, China) was selected as the flotation collector. No frothers and pH regulators were used. All reagents were used as received without further purification. Double-distilled water was used in all experiments and analytical measurements. All experiments were conducted at room temperature (22.5 ± 0.5 °C). The solid phase was separated by vacuum filtration using a circulating water vacuum pump (SHZ-3, Shanghai Yarong Biochemistry Factory, China). Whenever agitating was required, an agitator (IKA Eurostar 200, Ningbo Scientz Biotechnology Co. Ltd, Zhejiang, China) was used. A flotation machine (XFDIII-1.0 L, Jilin Prospecting Machinery Factory, Jilin, China) was used in flotation separation experiments.

### 2.2 Experimental procedures

In this work, a KCl single-mineral system was used as a mechanistic model to examine the intrinsic collector-KCl interactions and high-ionic-strength interfacial behavior in saturated KCl brine without confounding effects from other salts, gangue minerals, and fine particles. Its representativeness lies in preserving the target mineral, the saturated brine medium, and cationic collector adsorption on the mineral surface.

All the experiments were performed under controlled conditions: the temperature was maintained within ±0.5 °C, and all mass measurements were made to an accuracy of 0.01 g. After the completion of each flotation experiment, the resulting slurry was filtered to separate the solid and liquid phases. The floated and non-floated crystal fractions were collected, vacuum-filtered, dried, and weighed. To prepare the different concentration dispersions of ODA in water, ODA was heated to 70 °C. Once the ODA had melted, it was mixed with a certain quantity of distilled water containing HCl/HNO_3_/H_2_SO_4_/H_3_PO_4_ (also at 70 °C). The volume of HCl/HNO_3_/H_2_SO_4_/H_3_PO_4_ used was calculated to achieve a 1 : 1 M ratio of ODA to HCl/HNO_3_/H_2_SO_4_/H_3_PO_4_.

The flotation of salt mineral is typically carried out in their saturated mother liquors. Therefore, the preparation of these brines is of great importance. The KCl saturated brine was prepared simply by dissolving the respective salt crystals in water and stirring for 2 h (the remaining solid in the brine are indicative of saturation). The suspension was decanted overnight (at least 12 h) at room temperature to obtain a saturated brine for experimental use. Each flotation experiment was conducted under the same conditions: the pulp was stirred evenly for 5 minutes, the collector was added and then stirred evenly for another 5 minutes, and the flotation lasted for 5 minutes. For each group, three flotation tests were performed under identical conditions (ambient temperature, flotation duration, agitation speed, and bubbling frequency). The results are presented as the mean ± standard deviation (SD) of three independent experiments (*n* = 3). Given that the flotation experiments were performed on salt minerals, the samples were naturally air-dried after flotation. Forced dehydration was deliberately avoided, as it could induce evaporation and crystallization of the entrained mother liquor and thus compromise the interpretation of the FTIR and XPS spectra.

### 2.3 Analytical methods and characterizations

Particle size distribution (PSD) characteristics of the ODA-H samples were determined using a Malvern laser diffraction particle size analyzer (Malvern Instruments, Mastersizer 3000+) equipped with a wet dispersion unit. The PSD measurements were performed by dispersing a small aliquot of the ODA-H sample in the wet dispersion unit using water as the dispersant. The reported PSD therefore characterize the apparent particle-size distribution of ODA-H in a diluted aqueous suspension and do not represent the *in situ* particle-size distribution under saturated KCl brine flotation conditions. Each PSD measurement was performed once.

IR spectra were collected on a Thermo-Nicolet Nexus FTIR spectrometer (USA) equipped with a KBr crystal in the absorbance mode range from 400 to 4000 cm^−1^ with a resolution of 2 cm^−1^. All the measurements were made at room temperature.

X-ray photoelectron spectroscopy (XPS) was used to detect the adsorption states on KCl salt crystals before and after flotation treatment. All the samples were dried at ambient temperature, placed in sealed containers, and then transferred for measurement. XPS spectra were recorded using a Thermo Scientific K-Alpha photoelectron spectrometer (Thermo Fisher Scientific Inc., USA). Briefly, the sample was analyzed by a survey (wide) scan to identify the elements, followed by (narrow) high-resolution scans for each elemental region. The spectra were peak-fitted using the Avantage software package to gain quantitative information on the surface atomic concentrations. The spectra were charge corrected using the mainline of C 1s peak at the BE of 284.8 eV.

## 3 Results and discussion

### 3.1 ODA-H diversity and grain characteristics in low concentration

ODA is a typical cationic surfactant. In aqueous solutions, it does not exist as individual molecules or ions but instead displays a concentration-dependent self-assembly behavior. It tends to form spherical or rod-like micelles at low concentrations, transform into vesicles or layered liquid-crystalline structures at intermediate concentrations, and further aggregate into flocs or even macroscopic precipitates at high concentrations. At the concentration used in actual flotation, the ODA-H suspension appears milky and opaque at room temperature, and its microscopic morphology is difficult to analyze directly by conventional optical observation. Therefore, in this study, a series of diluted solutions were prepared with concentrations ranging from 10^−5^ to 10^−4^ mol L^−1^ (Fig. 1a–e), corresponding to 1/100 to 1/1000 of the actual ODA-H working concentration. This dilution facilitates observation of the aggregation morphology of ODA-H under transmitted light. Fig. 1f shows the overall morphology of ODA-H across this concentration gradient, clearly revealing that ODA-H is a white floc-like suspension with a network-like, fluffy structure. Although sample No. 5 has a concentration of 4.12 × 10^−4^ mol L^−1^, six times that of sample No. 1 (6.87 × 10^−5^ mol L^−1^), the characteristic white floc-like suspension is still very evident (as shown in Fig. 1f).

**Fig. 1:**
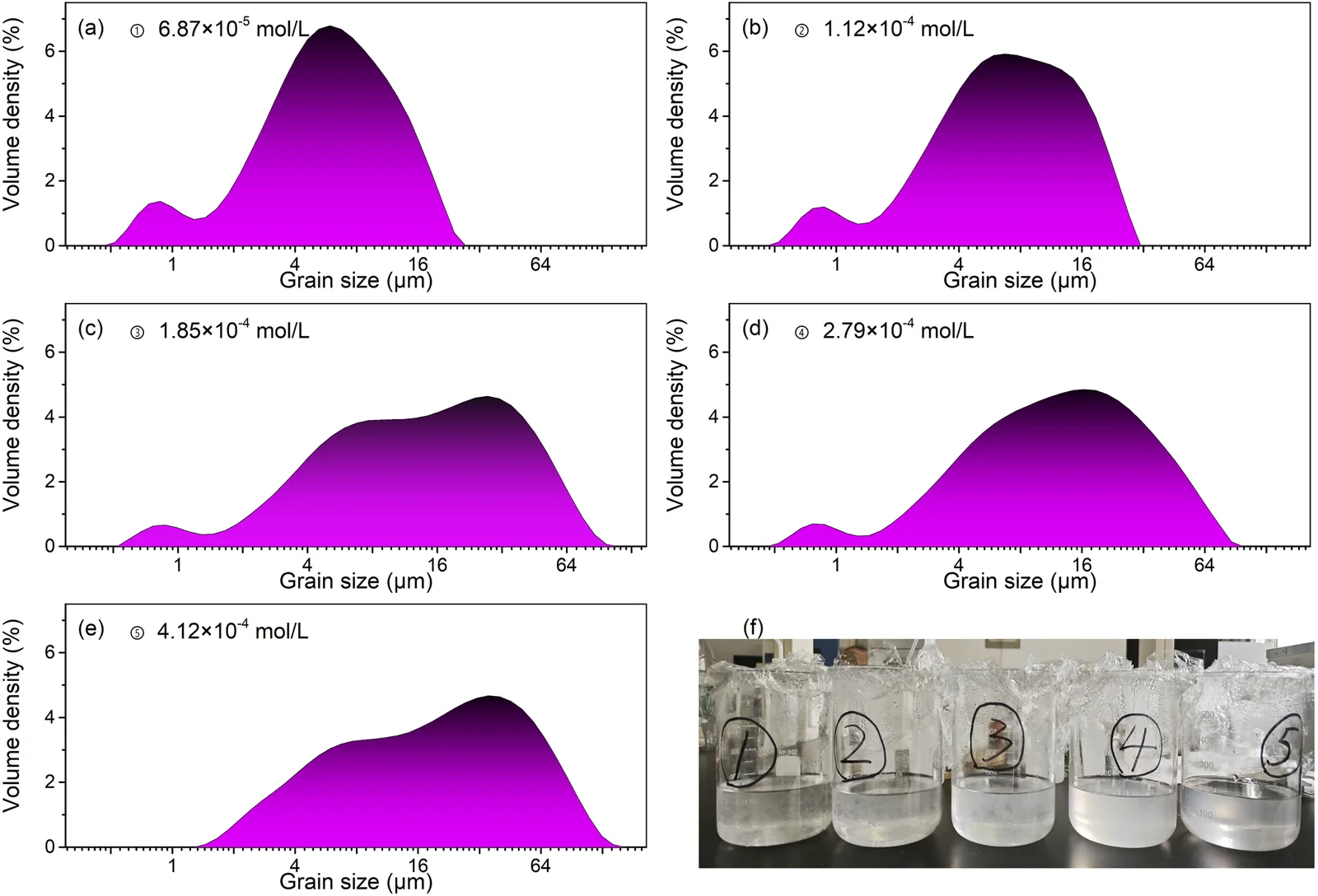
Grain size distribution characteristics of ODA-H at different concentrations: (a) 6.87 × 10^−5^ mol L; (b) 1.12 × 10^−4^ mol L^−1^; (c) 1.85 × 10^−4^ mol L^−1^; (d) 2.79 × 10^−4^ mol L^−1^; (e) 4.12 × 10^−4^ mol L^−1^; and (f) sample morphology.

The volume-based grain size distribution characteristics are shown in Fig. 1. In general, the characteristic grain size of the ODA-H flocculent suspension increases significantly with increasing concentration. As the ODA-H concentration increases from 6.87 × 10^−5^ mol L^−1^ to 4.12 × 10^−4^ mol L^−1^, the volume-average particle diameter (D[4,3]) rises significantly from 7.44 µm to 30.1 µm, indicating a more than 4-fold increase. In terms of the distribution pattern, the grain size distribution at each concentration follows a near-normal profile, but a weak secondary peak appears on the fine-grain side. This fine-grain secondary peak may be related to local inhomogeneity in dispersion or to the coexistence of different aggregate forms of ODA-H in acidic media. The small-grain peak (approximately 1–3 µm) corresponds to well-dispersed vesicles or thin lamellar micelles, which are thermodynamically stable or metastable small-scale aggregates, whereas the large-grain peak (approximately 8–15 µm) corresponds to flocculated networks or layered accumulations, which result from reversible inter-particle aggregation. As the ODA-H concentration increases, the Cl^−^ concentration in the system also rises because of the hydrochloric acid neutralization reaction, leading to a corresponding increase in ionic strength. The diffuse electric double layer on the grain surface is thereby compressed, and the electrostatic repulsion barrier is significantly reduced. According to DLVO theory, when the repulsive force weakens to the point where it can no longer stabilize the dispersed particles, reversible flocculation occurs at the secondary minimum, *i.e.*, aggregation induced by overlap of the diffuse double layers, resulting in the formation of loosely structured but voluminous flocs.

To further quantify the grain size distribution characteristics of the ODA-H flocculent suspension at different concentrations, the characteristic grain size parameters of each sample, namely D10, D50, D90, and D100, were statistically analyzed, and the distribution span index [(D90–D10)/D50] was calculated (Fig. 2a–f). As the ODA-H concentration increased from 6.87 × 10^−5^ mol L^−1^ to 4.12 × 10^−4^ mol L^−1^, D10, D50, and D90 all increased monotonically. The D50 increased from approximately 6.17 µm at 6.87 × 10^−5^ mol L^−1^ to approximately 21.4 µm at 4.12 × 10^−4^ mol L^−1^ (an increase by a factor of nearly 3.5). This trend is consistent with the rightward shift of the main peak in the volume-based particle size distribution shown in Fig. 1. The increase in D90 was even more pronounced, indicating that the contribution of the coarse end to the large-size fraction was progressively amplified as flocculation deepened. D100 (maximum grain size) also exhibited a simultaneous upward trend at each concentration, reflecting the continuous expansion of the upper size limit of large flocs with increasing concentration. The [(D90–D10)/D50] value, as a direct measure of the width of the grain size distribution, reveals the evolution of dispersion homogeneity in the system. At low concentrations (6.87 × 10^−5^ and 1.12 × 10^−4^ mol L^−1^), the span [(D90–D10)/D50] remained relatively low (2.09 and 2.23, respectively; see Fig. 2f), indicating a relatively narrow particle size distribution. This is consistent with the narrow peaks and near-normal distribution profiles shown in Fig. 1a and b. As the concentration further increased (1.85 × 10^−4^ mol L^−1^ and above), the span increased markedly to a range of 2.96–3.04, reflecting a pronounced broadening of the particle size distribution caused by the formation of large flocs. These quantitative results confirm the visual observations in Fig. 1c–e, demonstrating the intensifying bimodal character and the expansion of the distribution range.

**Fig. 2:**
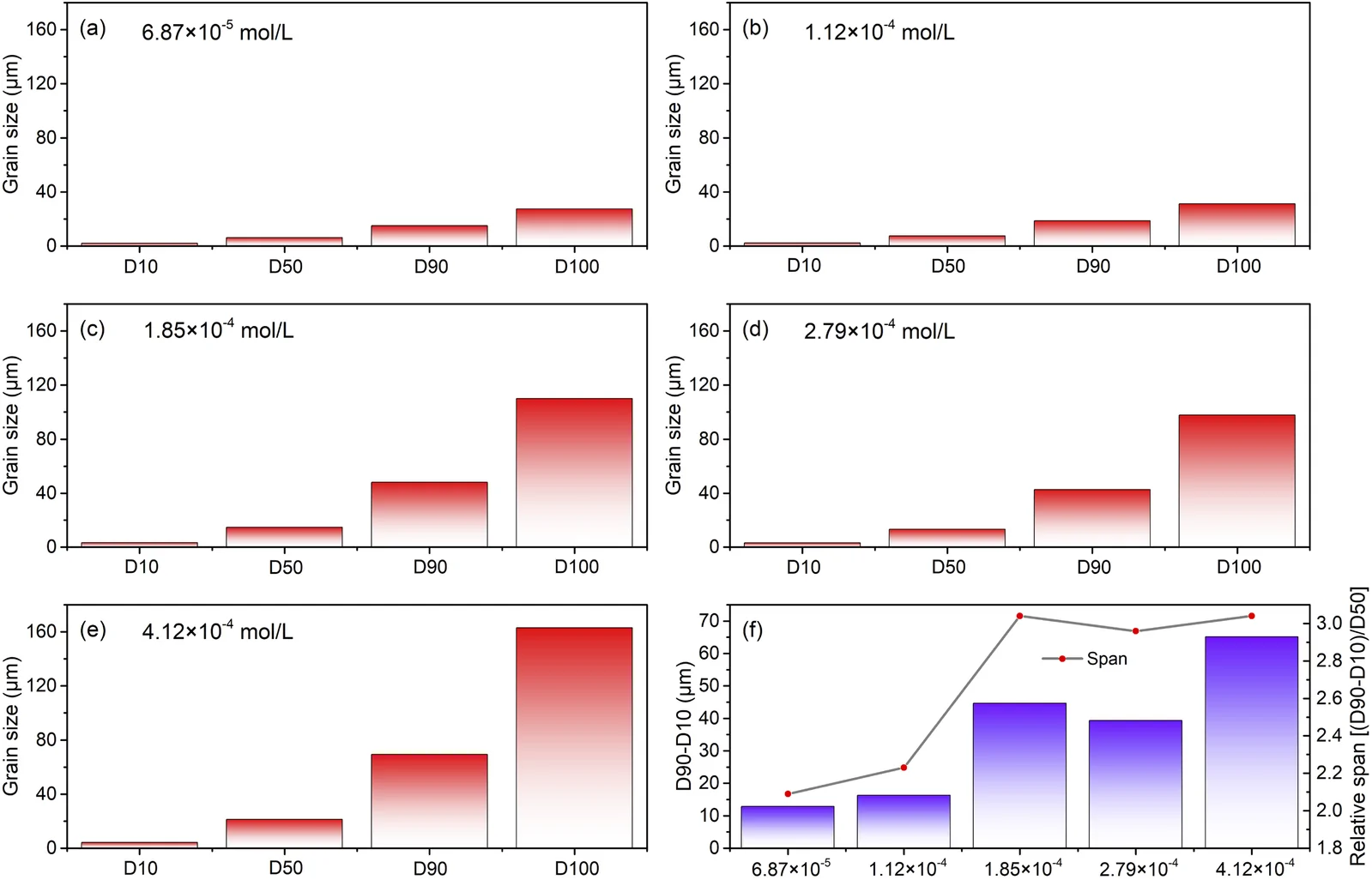
Grain size parameters distribution characteristics of ODA-H at different concentrations: (a) 6.87 × 10^−5^ mol L^−1^; (b) 1.12 × 10^−4^ mol L^−1^; (c) 1.85 × 10^−4^ mol L^−1^; (d) 2.79 × 10^−4^ mol L^−1^; (e) 4.12 × 10^−4^ mol L^−1^; and (f) distribution span index.

### 3.2 ODA-H diversity and grain characteristics in high concentration

Within the conventional usage concentration range of ODA-H (around 1 × 10^−2^ mol L^−1^, 0.3 wt%), three concentration-gradient samples were prepared: P-1 (7.38 × 10^−3^ mol L^−1^, 0.2 wt%), P-2 (1 × 10^−2^ mol L^−1^, 0.3 wt%), and P-3 (3.71 × 10^−2^ mol L^−1^, 1.0 wt%). Their volume-based grain size distributions are shown in Fig. 3a–c, and their macroscopic appearance is presented in Fig. 4h. All samples appeared as white, flocculent, emulsion-like dispersed suspensions. As shown in Fig. 3a–c, the grain size distributions of the ODA-H flocculent suspensions cover a broadly similar range, mainly from about 4 to 256 µm. However, with increasing ODA-H concentration, the volume-average particle diameter (D[4,3]) decreases from 55.7 µm (P-1) to 31.4 µm (P-2), before increasing slightly to 40.5 µm (P-3). This indicates that the grain size of the suspension decreases slightly with increasing ODA-H concentration. Using a fixed ODA-H concentration of 3.71 × 10^−2^ mol L^−1^ (1.0 wt%), the influence of different inorganic acids (HCl, H_2_SO_4_, HNO_3_, and H_3_PO_4_) on the floc grain size was further investigated. The corresponding volume-based grain size distributions are shown in Fig. 3c–f. The type of acid anion significantly affects the grain size distribution of the flocculated dispersion. The HCl system (P-3, Fig. 3c) exhibits a typical unimodal distribution, with grain sizes concentrated between approximately 4 and 256 µm and a median diameter (D50) of 25.6 µm. When H_2_SO_4_ is used (P-4, Fig. 3d), intense agglomeration markedly shifts the main peak to approximately 64–512 µm, dramatically increases the median diameter (D50) of 212 µm, and makes the distribution extremely broad. In addition, during preparation, ODA was observed to dissolve and disperse poorly in hot water containing H_2_SO_4_. The HNO_3_ system (P-5, Fig. 3e) shows a clear bimodal distribution, with a smaller grain peak at approximately 4–16 µm and a larger aggregate fraction at approximately 32–256 µm and a median diameter (D50) of 12.9 µm, indicating an uneven grain size distribution. The H_3_PO_4_ system (P-6, Fig. 3f) retains a unimodal shape, but the particle sizes are slightly larger than those in the HCl system, with the main peak centered roughly between 32 and 128 µm and a median diameter (D50) of 35.9 µm. In summary, the grain size of the ODA-H flocculent dispersion varies greatly depending on the anion type. The sulfuric acid system produces the most severe agglomeration, the nitric acid system tends to form a bimodal dispersion, and the phosphoric acid system yields slightly coarser flocs than the hydrochloric acid system.

**Fig. 3:**
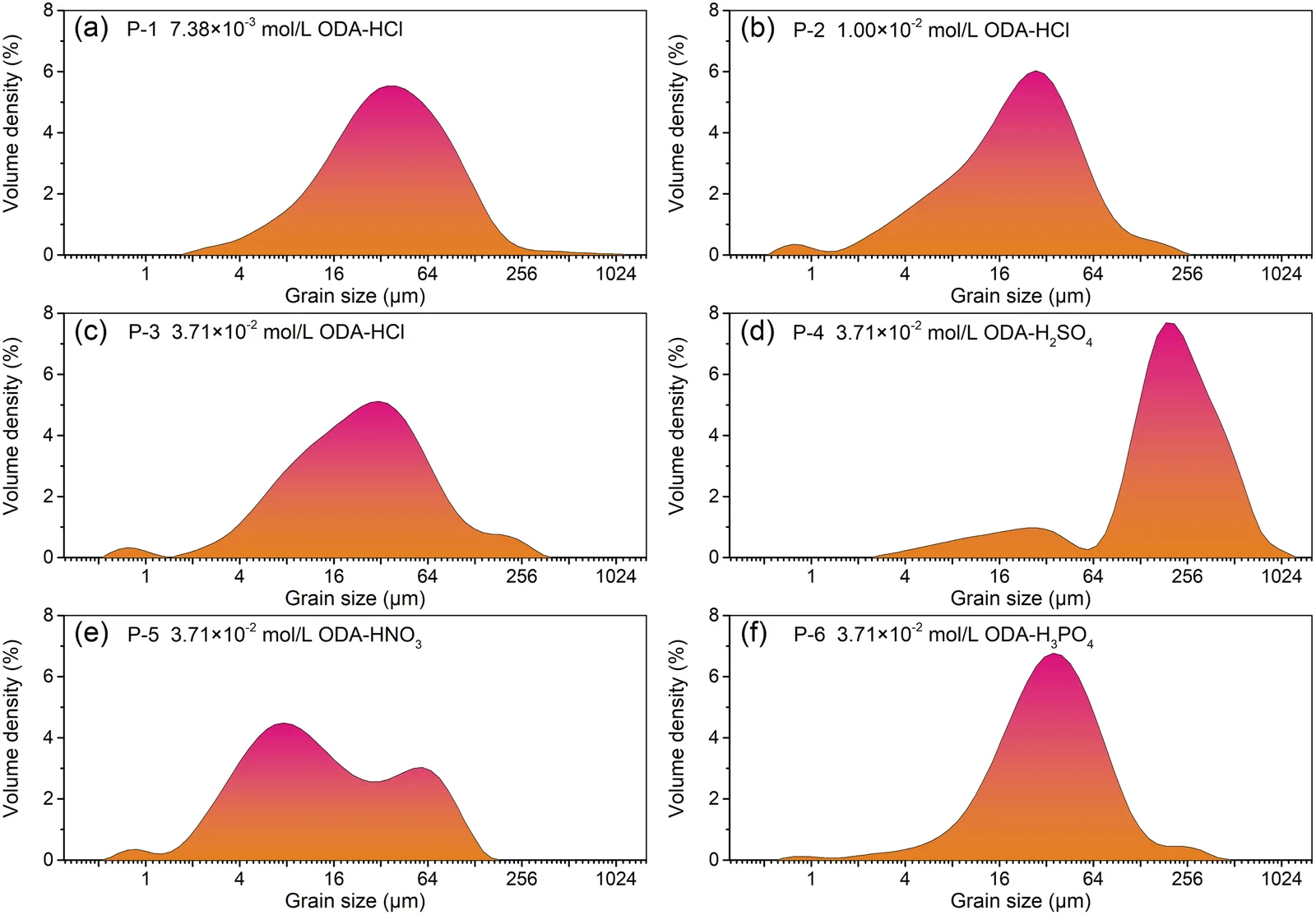
Grain size distribution characteristics of ODA-H at different concentrations (a) 7.38 × 10^−3^ mol L^−1^; (b) 1.12 × 10^−2^ mol L^−1^; (c) 3.71 × 10^−2^ mol L^−1^; and different inorganic acids (d) H_2_SO_4_; (e) HNO_3_; (f) H_3_PO_4_.

**Fig. 4:**
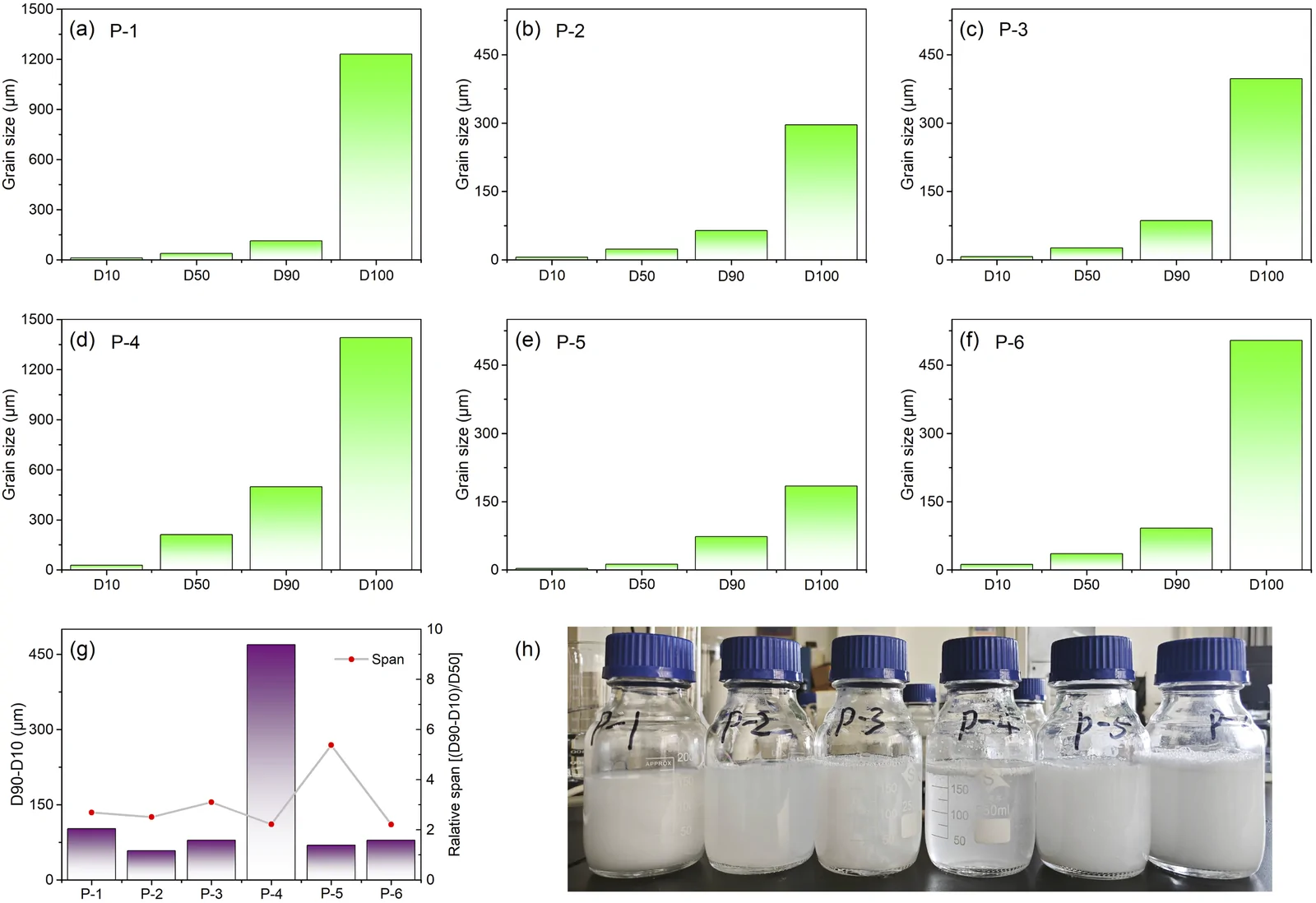
Grain size parameters distribution characteristics of ODA-H at different concentrations: (a) 7.38 × 10^−3^ mol L^−1^; (b) 1.12 × 10^−2^ mol L^−1^; (c) 3.71 × 10^−2^ mol L^−1^; and different inorganic acids (d) H_2_SO_4_; (e) HNO_3_; (f) H_3_PO_4_, and (g) distribution span index; (h) sample morphology.

Fig. 4a–f presents the characteristic particle size parameters (D10, D50, D90, and D100) of each sample. For the ODA-H concentration gradient (P-1, P-2, P-3), although the main peak shifts slightly toward smaller sizes with increasing concentration (Fig. 4a–c), the D100 of P-1 is abnormally high at approximately 1200 µm, indicating that exceptionally large aggregates are locally present even though the main peak remains concentrated between 30 and 40 µm at the low concentration of 0.2 wt%. In contrast, the D100 values of P-2 and P-3 decrease to approximately 300 and 400 µm, respectively, reflecting a more convergent size distribution. Among the different acid systems at a fixed concentration (P-3, P-4, P-5, P-6), the differences in grain size are more pronounced. Fig. 4d shows that the P-4 system prepared with sulfuric acid (H_2_SO_4_) undergoes intense agglomeration, as evidenced by a substantial increase in D50 to approximately 200 µm and in D90 and D100 to approximately 600 and 1400 µm, respectively. This severe agglomeration is attributed to the poor solubility of ODA in the sulfuric acid system. Fig. 4g presents the distribution span index of each sample. The purple bars represent the absolute span (D90–D10, in the same units as grain size), where P-4 stands out with a value close to 450 µm, indicating highly non-uniform aggregate sizes. The red line shows the relative span [(D90–D10)/D50]. P-5 (nitric acid system) exhibits the highest relative span (∼5.5). In spite of its moderate absolute value, it confirms the bimodal distribution observed in Fig. 3e and indicates poor particle size uniformity. In contrast, although P-3 exhibits a broader span (3.09), the spans of P-2 (2.51) and P-6 (2.21) are relatively lower, indicating a more uniform and narrowly distributed floc size in the conventional hydrochloric acid and phosphoric acid systems compared to the severely aggregated samples. Fig. 4h presents the macroscopic appearance of samples P-1 to P-6, all of which are white, flocculent, emulsion-like dispersed suspensions.

The critical micelle concentration (CMC) of ODA/ODA·HCl has been reported to be in the range of 10^−4^–10^−3^ mol L^−1^ in aqueous solution,^41^ and to decrease to approximately 10^−5^ mol L^−1^ at high ionic strength.^41,42^ The actual flotation concentration employed here (10^−2^ mol L^−1^) is therefore well above the CMC, indicating that ODA·HCl exists predominantly in the form of micellar aggregates under flotation conditions. In contrast, the dilute systems used for morphological observation (10^−5^–10^−4^ mol L^−1^) lie near or below the CMC, where the surfactant is present mainly as monomers and pre-micellar flocs. This distinction accounts for the divergent aggregation states observed under the two concentration regimes. It further supports the interpretation that the concentration-dependent evolution of the PSD, characterized by initial floc growth followed by floc refinement at higher concentrations, originates from the transition from pre-micellar flocculation to micelle-dominated aggregation. The detailed experimental data are provided in the SI (Table S1).

### 3.3 Relationship between ODA-H concentration and KCl flotation recovery

The flotation results are presented in Fig. 5 (*n* = 3 independent experiments), and the detailed experimental data and error calculations are provided in the SI (Table S2–S8). Fig. 5a illustrates the coupled effects of ODA-H concentration on KCl flotation recovery and collector dosage, revealing a clear inverse relationship. As the ODA-HCl concentration increases, KCl recovery rises rapidly at first and then more gradually. KCl recovery increases sharply from approximately 87.5% at 7.4 × 10^−3^ mol L^−1^ to over 94% at 1.6 × 10^−2^ mol L^−1^, ultimately reaching a peak of about 95.8% in the high-concentration range. Meanwhile, collector dosage decreases markedly, dropping from about 11.5 mL/100 g raw ore at low concentrations to 1.5 mL/100 g raw ore at high concentrations, indicating that high-concentration reagents exhibit much higher volumetric utilization efficiency. Overall, maintaining the ODA-HCl concentration within 1.0 × 10^−2^ to 1.6 × 10^−2^ mol L^−1^ represents the optimal operating window, corresponding to a collector dosage of approximately 6–8 mL/100 g raw ore. Under these conditions, a stable flotation recovery exceeding 94% can be achieved together with economical reagent consumption, providing an optimal balance between flotation performance and process economics. It should be noted that while the absolute maximum recovery (95.89%) is achieved at a higher concentration (1.00 wt%), this requires a fourfold increase in reagent dosage for a marginal recovery gain of only 2.36%. Therefore, the concentration range of 1.0 × 10^−2^–1.6 × 10^−2^ mol L^−1^ is defined as the optimal condition for best overall process efficiency, aligning with typical industrial practices (0.25–0.50 wt%).

**Fig. 5:**
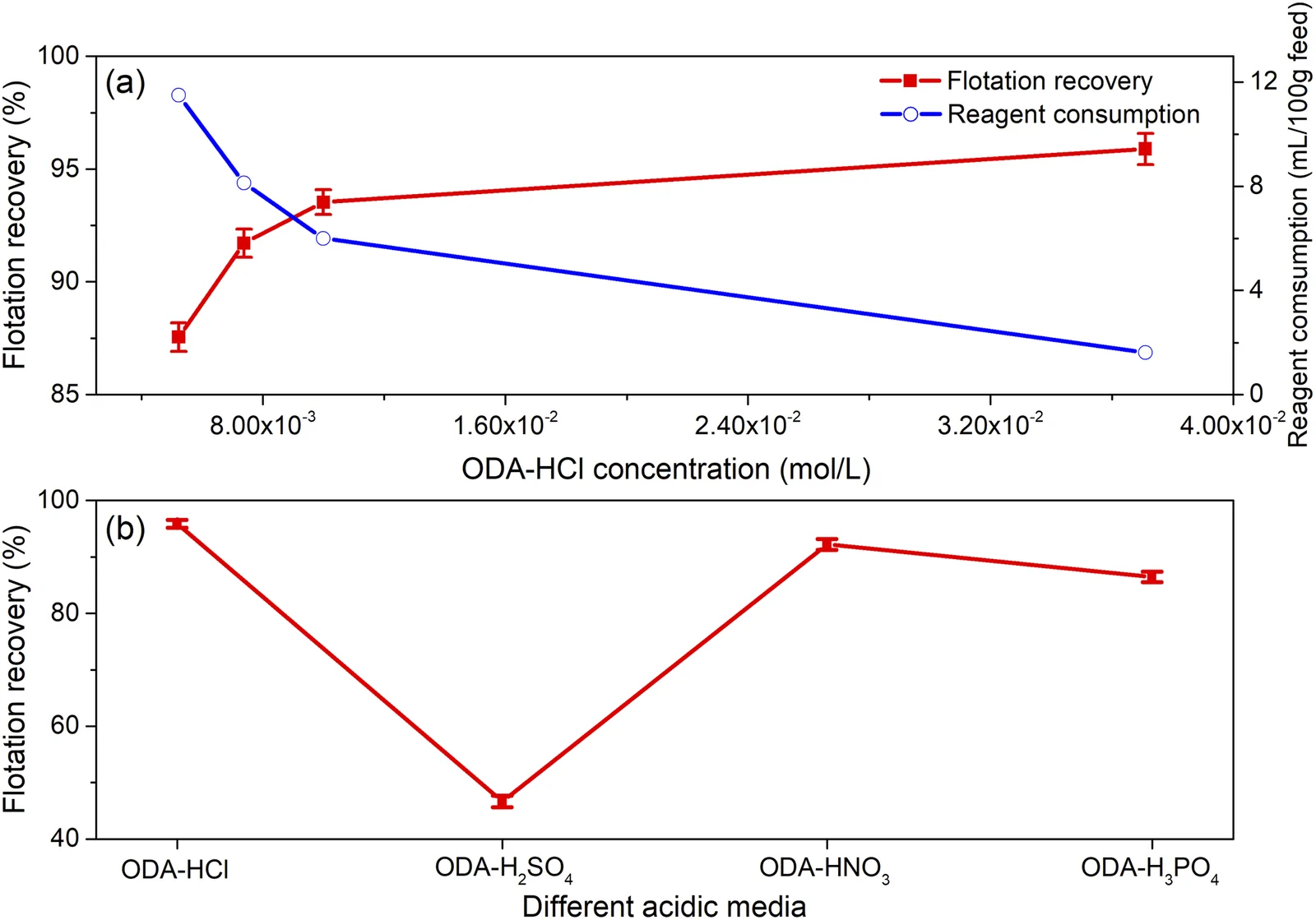
(a) Coupled effects of ODA-H concentration on KCl flotation recovery and collector dosage; (b) KCl flotation recovery under different inorganic acid ODA-HX system.

Based on the grain size data for the ODA-H concentration gradient samples (Fig. 3a–c and Fig. 4a–c, P-1 to P-3), the main peak diameter of the flocculent material in the low-concentration sample P-1 (7.38 × 10^−3^ mol L^−1^) is 38.1 µm, but its D100 reaches an abnormally high value of approximately 1200 µm, indicating the presence of numerous extremely non-uniform large clusters. As the concentration increases to P-2 (1 × 10^−2^ mol L^−1^) and P-3 (3.71 × 10^−2^ mol L^−1^), the main peak of the size distribution gradually shifts to 31.1 µm and 25.6 µm, respectively, while the distribution span index significantly to below 2.0 (Fig. 4g). This indicates that at higher concentrations, ODA-H forms flocs that are smaller, more concentrated, and more uniform. Meanwhile, salt flotation is strictly constrained by the saturated mother liquor concentration. As shown in Fig. 5a, the dosage varies from 11.5 mL/100 g raw ore (low concentration) to 1.5 mL/100 g raw ore (high concentration), meaning an additional 10 mL of water per 100 g of ore is introduced at room temperature. This extra water could dissolve up to approximately 3.42 g of KCl. This value serves as an estimated maximum (upper-bound) KCl dissolution loss based on the solubility of KCl at 25 °C. It should be noted that this is a theoretical estimate rather than a direct experimental measurement, assuming the added water is initially KCl-free and the liquid eventually reaches saturation. Consequently, such water introduction may lead to a potential loss of KCl resources. This microscopic grain size difference of ODA-H is strongly coupled with the macroscopic flotation performance and water-induced dissolution loss. At the low-concentration stage, the flocculent material not only contains large abnormal agglomerates and exhibits a very wide size span, but also achieves a flotation recovery of only 87.5%; moreover, the higher reagent dosage (11.5 mL/100 g raw ore) further destabilizes the saturated mother liquor system and aggravates potassium salt dissolution. In contrast, at the high-concentration stage, the flocs are finer and more uniform, and only a very small reagent volume (1.5 mL/100 g raw ore) is needed to achieve a high flotation recovery of 95.8%. This minimizes the disturbance of added water to the mother liquor balance and effectively protects KCl minerals from dissolution loss.

Fig. 5b presents KCl flotation recovery under different inorganic acid ODA-HX system. All four acid systems were tested at the same ODA-H concentration of 3.71 × 10^−2^ mol L^−1^ and a dosage of 1.62 mL/100 g raw ore, yet they exhibited markedly different flotation responses. The ODA-HCl system achieved the highest KCl recovery (>94%), the ODA-HNO_3_ and ODA-H_3_PO_4_ systems showed intermediate performance, and the ODA-H_2_SO_4_ system underwent a precipitous decline, with recovery dropping sharply to approximately 47%. These macroscopic differences are essentially attributable to variations in the hydration characteristics of the anions and their competitive adsorption behavior at the interface. Specifically, Cl^−^, being the inherent anion of the KCl lattice, does not undergo significant competitive adsorption in the aqueous solution and helps preserve the negative surface charge of the mineral, thereby promoting the firm electrostatic adsorption of prorogated ODA (R-NH_3_^+^) onto the KCl surface to form a hydrophobic film, which accounts for the highest recovery. In contrast, SO_4_^2−^, as a divalent strongly hydrated anion, readily forms hydrophilic ion pairs with the amino groups of ODA and builds a thick hydration layer on the mineral surface, severely hindering the film-forming ability of the collector and ultimately leading to the complete failure of flotation.

### 3.4 FTIR characteristic on KCl surface during flotation

To investigate the adsorption states of ODA on the KCl surface after flotation with different ODA-H concentrations, FTIR analysis was carried out on dynamic flotation products obtained at ODA-H concentrations of 7.38 × 10^−3^ mol L^−1^ (P-1), 1 × 10^−2^ mol L^−1^ (P-2), and 3.71 × 10^−2^ mol L^−1^ (P-3), as well as on static immersion products prepared at the corresponding concentrations (XPS-1, XPS-2, and XPS-3). The results are shown in Fig. 6 and 7.

**Fig. 6:**
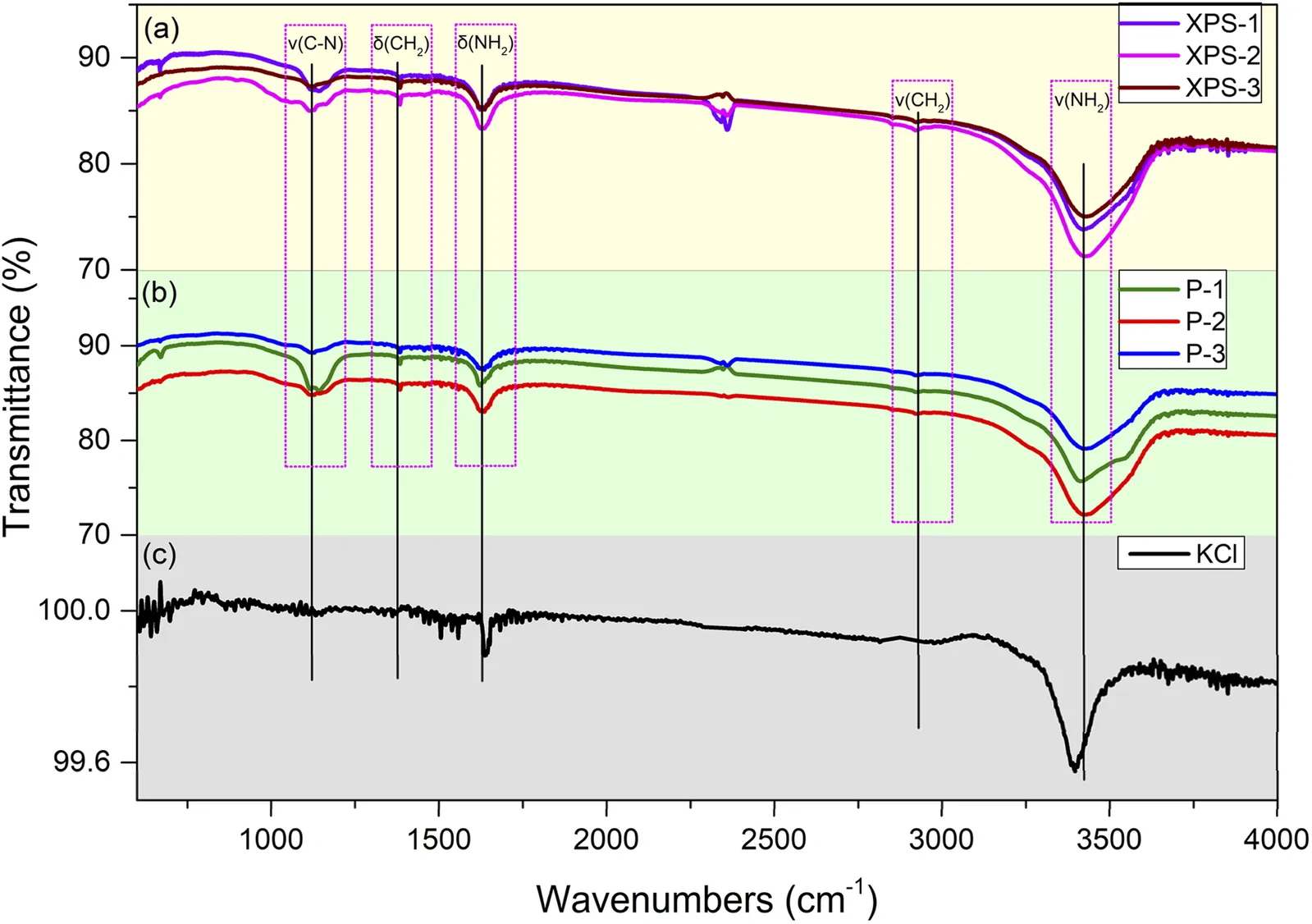
FTIR characteristics of ODA on the KCl surface (a) static immersion; (b) dynamic flotation, and (c) untreated KCl.

**Fig. 7:**
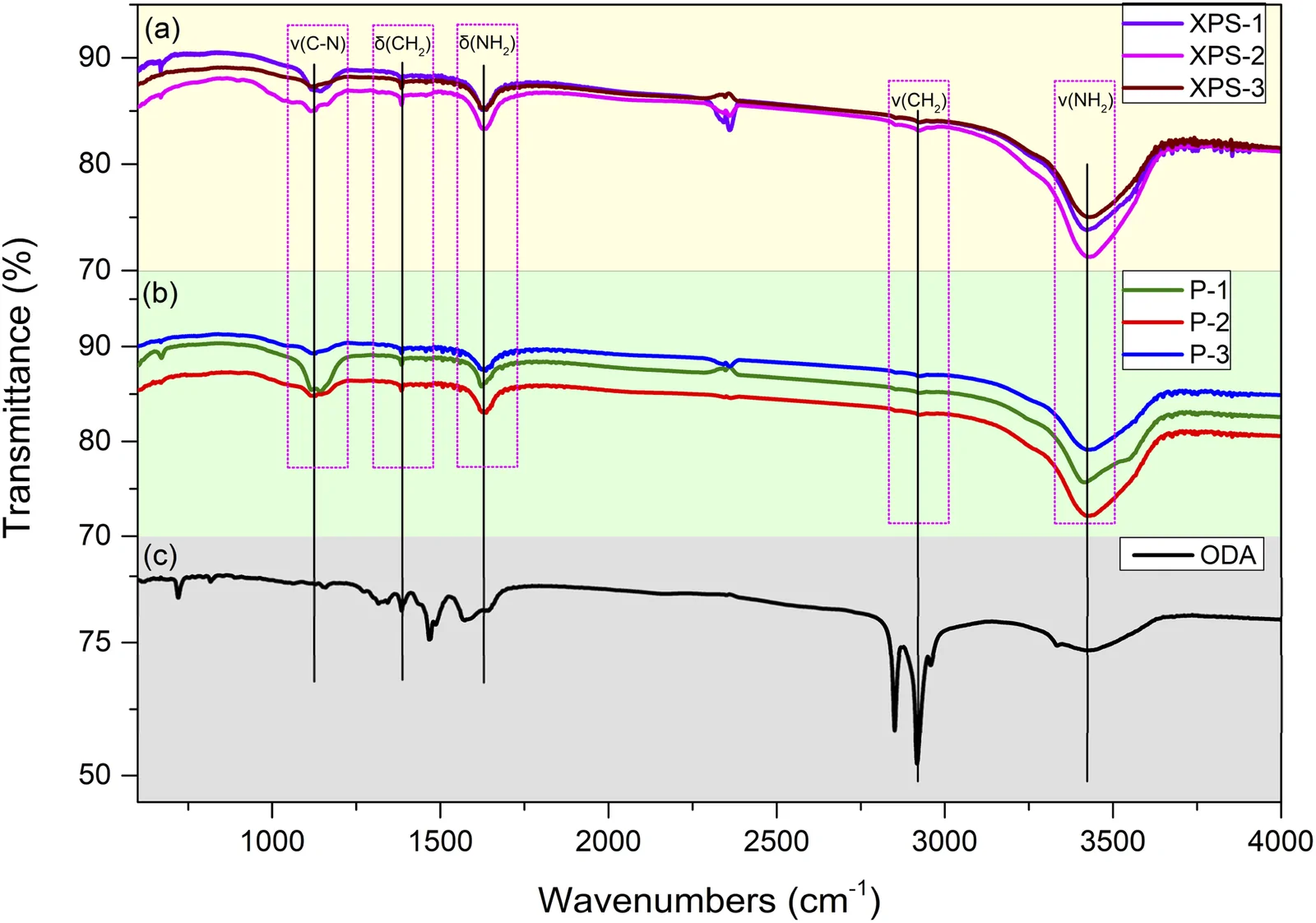
FTIR characteristics of ODA on the KCl surface (a) static immersion; (b) dynamic flotation, and (c) ODA.

Fig. 6c shows the spectrum of untreated KCl, which exhibits only weak absorption from water molecules or hydroxyl groups. In contrast, all samples in Fig. 6(a) and (b) display characteristic functional group peaks of ODA-H at approximately 3400–3500 cm^−1^ (*γ*(NH_2_)), 2850–2950 cm^−1^ (*γ*(CH_2_)), 1600 cm^−1^ (*δ*(NH_2_)), and 1460 cm^−1^ (*δ*(CH_2_)), confirming successful adsorption of the collector onto the KCl surface. The infrared absorption intensities also vary with ODA-H concentration. In Fig. 6b, P-2 (red curve) shows the lowest transmittance (*i.e.*, highest absorbance) in the N–H and C–H regions, P-1 (green curve) shows higher transmittance, and P-3 (blue curve) lies between them. This indicates that at the intermediate concentration (1 × 10^−2^ mol L^−1^, P-2), the adsorption density and effective interaction of ODA-H on the particle surface reach a pronounced maximum. This is consistent with the particle size data, where P-2 exhibits the most concentrated size distribution (D100 ≈ 300 µm, span ≈ 2.0). Under these optimal adsorption conditions, the collector molecules form a highly uniform and dense hydrophobic layer on the mineral surface, preventing the non-uniform aggregation and low recovery associated with P-1 (weak adsorption, high transmittance) and avoiding the marginal improvements achieved only at excessively high concentrations (P-3). A comparison of static equilibrium adsorption (Fig. 6a) and dynamic flotation adsorption (Fig. 6b) reveals distinct behaviors. In the 12-hours static immersion experiments, the intensity of characteristic peaks increases monotonically with ODA-H concentration, with XPS-3 (highest concentration) exhibiting the greatest total adsorption. In contrast, under real flotation conditions, the adsorption intensity follows a non-linear order: P-2 (intermediate concentration) > P-3 (high concentration) > P-1 (low concentration). P-2 displays markedly higher interfacial adsorption activity within a short time, reflecting the special kinetics of flotation. At excessive ODA-H concentrations, abundant free micelles form in solution, reducing the effective monomer concentration available for interfacial adsorption and thereby lowering surface coverage efficiency during dynamic flotation. In contrast, P-2, at an appropriate concentration, achieves high coverage and forms a stable “semi-gel” structure on the KCl surface. These microscopic adsorption differences correlate strongly with macroscopic flotation recovery and particle size distribution. The high dynamic adsorption activity of P-2 corresponds to the most uniform particle size distribution (D100 ≈ 300 µm, span ≤ 2.0), as the uniform and dense adsorption prevents the severe non-selective aggregation observed for P-1 (weak adsorption, D100 ≈ 1200 µm with very large agglomerates) and avoids the problems associated with high-concentration P-3, where excessive micelles cause over-stabilized froth and reduced effective adsorption efficiency. Consequently, P-2 achieves a high flotation recovery exceeding 94%.

Fig. 7c presents the FTIR spectrum of pure ODA (octadecylamine) for comparison, further confirming the adsorption behavior of ODA-HCl on KCl. In the spectrum of pure ODA (Fig. 7c), the C–H stretching vibrations of the long-chain methylene groups (approximately 2850–2950 cm^−1^) are extremely sharp and intense, and the characteristic N–H absorption appears near 3300–3400 cm^−1^. In contrast, the adsorbed samples (Fig. 7a and b) show significant peak broadening and shifts, particularly near 1600 cm^−1^ and 3400–3500 cm^−1^. These changes indicate that ODA is protonated by hydrochloric acid to form ODA·HCl, which adsorbs onto the KCl surface as a cationic surfactant through electrostatic attraction or hydrogen bonding, while the conformation of the hydrophobic long carbon chains is altered by interfacial interactions.

As shown in Table 1, compared with pure ODA, the characteristic peaks assigned to the polar groups exhibit noticeable red shiftschemafter adsorption. Specifically, the *δ*(NH_2_) bending vibration shifts from 1643 cm^−1^ to the range of 1622–1636 cm^−1^, and the *ν*(C–N) stretching vibration shifts from 1158 cm^−1^ to 1123–1143 cm^−1^. These shifts indicate that the electron cloud density around the C–N bond and amino groups is redistributed due to the interaction (such as electrostatic attraction or hydrogen bonding) between the –NH_2_ groups and the KCl surface. In contrast, the characteristic peaks of the alkyl chains (*ν*(CH_2_) at ∼2917 cm^−1^ and *δ*(CH_2_) at ∼1384 cm^−1^) show only minor fluctuations (±9 cm^−1^), suggesting that the hydrophobic tails remain largely undisturbed and are oriented outward.

**Table 1:** FTIR characteristic peak positions and their corresponding shifts of pure ODA, untreated KCl, and ODA adsorbed on KCl surface^a^

Condition	Sample	*ν*(C–N) cm^−1^	*δ*(CH_2_) cm^−1^	*δ*(NH_2_) cm^−1^	*ν*(CH_2_) cm^−1^	*ν*(NH_2_) cm^−1^
Pure ODA		1158	1384	1643	2917	3424
Untreated KCl		—	—	—	—	—
After ODA adsorption on KCl	XPS-1	1142 (−16)	1384 (0)	1636 (−7)	2923 (+6)	3422 (−2)
Static immersion	XPS-2	1143 (−15)	1384 (0)	1627 (−16)	2924 (+7)	3426 (+2)
	XPS-3	1139 (−19)	1384 (0)	1636 (−7)	2926 (+9)	3431 (+7)
After ODA adsorption on KCl	P-1	1142 (−16)	1386 (+2)	1622 (−21)	2926 (+9)	3416 (−8)
Dynamic flotation	P-2	1123 (−35)	1386 (+2)	1636 (−7)	2926 (+9)	3423 (−1)
	P-3	1131 (−27)	1384 (0)	1635 (−8)	2924 (+7)	3422 (−2)

a The values in parentheses indicate the peak shifts (Δ*ν* = *ν*_adsorbed_ − *ν*_pureODA_). A negative value indicates a red shift to a lower wavenumber.

### 3.5 XPS characteristic on KCl surface during flotation

Fig. 8a presents the survey spectrum of the flotation KCl product surface. In addition to the K and Cl elements inherent to the KCl matrix, N and a substantial amount of C were also detected. The appearance of N directly confirms that the amine-bearing collector (ODA-H) has successfully adhered to the KCl surface, while the intense C signal originates primarily from the long carbon chains of ODA. A small amount of O was also observed, most likely from adsorbed water, hydroxyl groups, or trace oxidized impurities introduced during sample preparation or measurement; this does not affect the main adsorption analysis. The C 1s high-resolution spectrum in Fig. 8b exhibits a single, intense C–C/C–H peak at approximately 284.8 eV, with no significant C–O or C=O contributions, indicating that the detected carbon corresponds to the long alkyl chains of ODA. K 2p shows the typical doublet (2p_1/2_ and 2p_3/2_) with a spin–orbit splitting consistent with the KCl lattice (Fig. 8c), while the Cl 2p doublet also matches the standard chemical state of chloride ions (Fig. 8d). No significant chemical shifts are observed, indicating that ODA-H adsorption on KCl is dominated by physical or electrostatic interactions and does not disrupt the KCl crystal structure or form new strong covalent bonds. This is highly consistent with the classical flotation theory in which cationic surfactants act on the negatively charged KCl surface through electrostatic attraction.

**Fig. 8:**
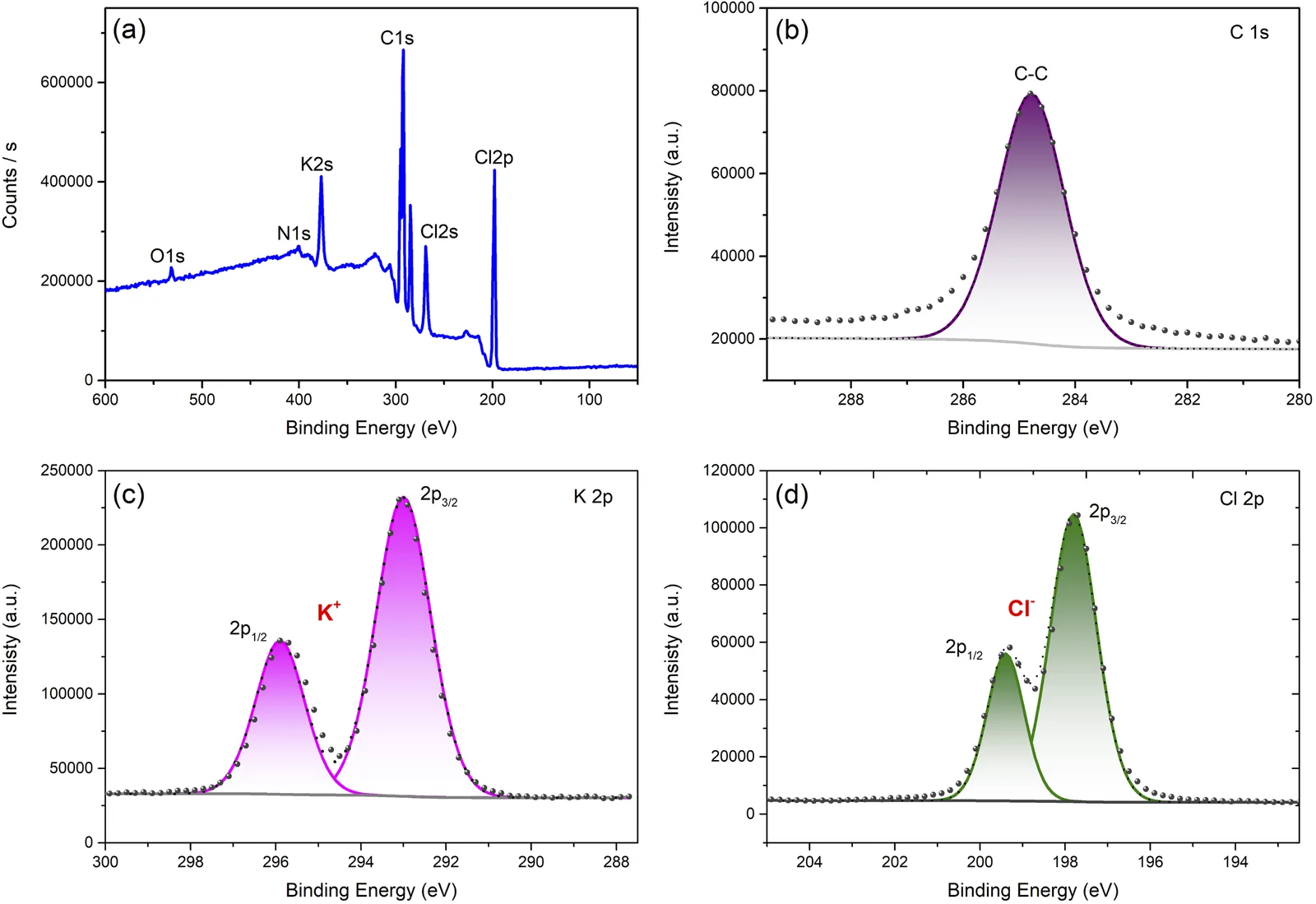
XPS spectra of KCl after ODA-H adsorption: (a) survey, (b) C 1s, (c) K 2p, and (d) Cl 2p.

Fig. 9 shows the N 1s high-resolution spectrum, which exhibits a fitted peak at a binding energy of approximately 399.5 eV, assigned to C–N. Because the C : N ratio in ODA-H is 18 : 1, the N 1s signal is weaker and noisier than the C 1s spectrum; nevertheless, the fitted peak corresponds well to the chemical bonding between the amino group (–NH_2_/–NH_3_^+^) and the long-chain alkyl skeleton in the octadecylamine molecule. This result conclusively demonstrates that intact ODA molecules are retained on the surface of the flotation concentrate, without chemical bond cleavage or degradation. Moreover, the binding energy position is characteristic of an organic amine rather than an inorganic salt complex, further supporting the conclusion that ODA-HCl interacts with the KCl surface mainly through electrostatic physical adsorption rather than deep chemical reconstruction.

**Fig. 9:**
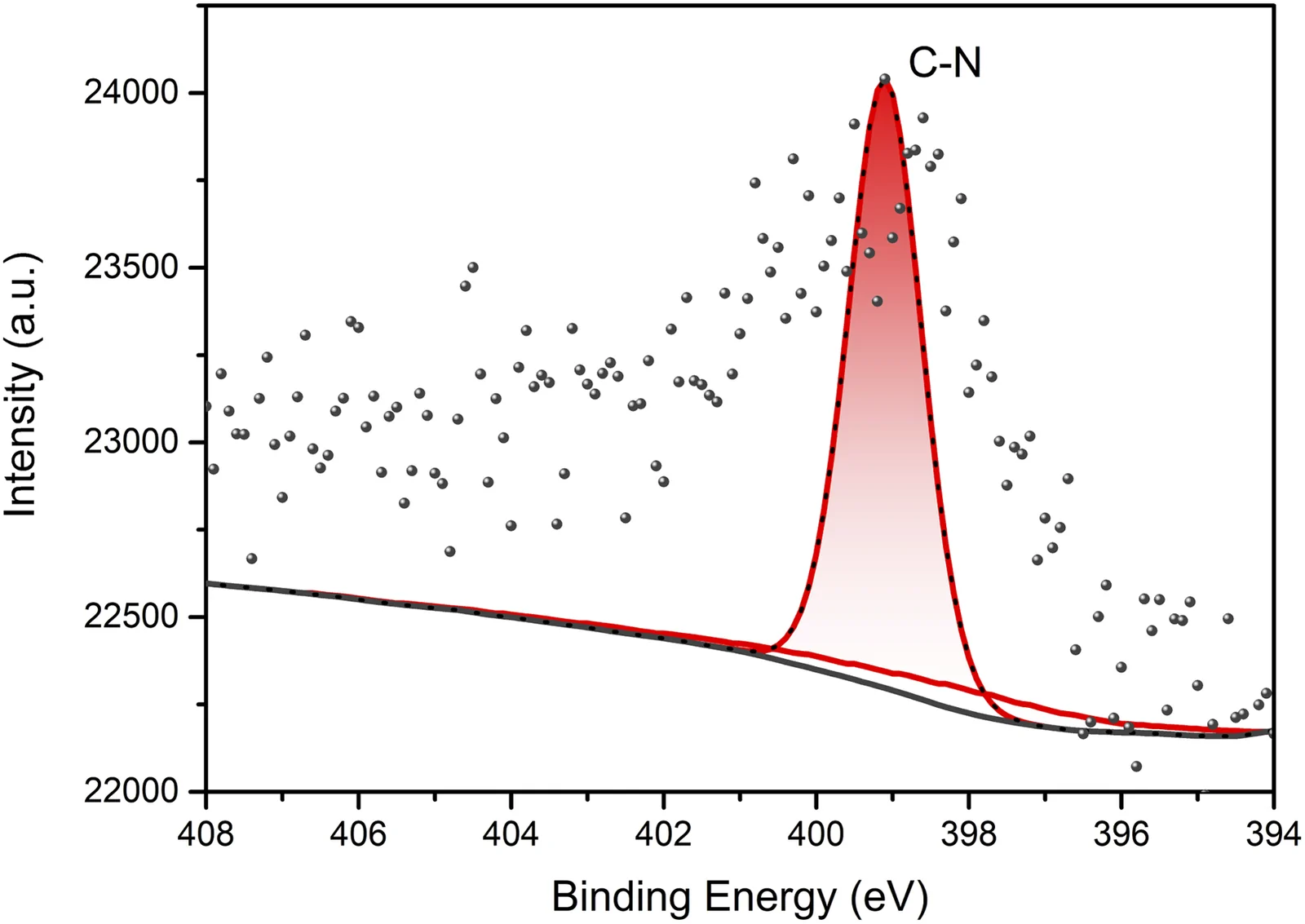
N 1s XPS spectrum of KCl after ODA-H adsorption.

The XPS quantitative analysis (Table 2) corroborates the successful adsorption of the ODA-H collector onto the KCl surface. Upon ODA-H treatment, the surface atomic percentage of C 1s surges from 16.44% to 49.15%, accompanied by a distinct increase in N 1s from 0.71% to 2.49%. The appearance of this pronounced nitrogen signal is a direct fingerprint of the amino groups within the ODA-H molecules, while the high initial carbon content on the pristine KCl is attributed to inevitable adventitious carbon contamination from ambient exposure. Conversely, the relative contents of K 2p and Cl 2p decrease significantly (from 26.10% to 16.81% and 25.12% to 17.23%, respectively). This phenomenon is primarily attributed to the attenuation effect of the newly formed organic overlayer, which blocks the photoelectrons emitted from the underlying KCl substrate, thereby further confirming the existence of a continuous surface coating. Most importantly, the C/N atomic ratio of the adsorbed layer is measured at 19.74, which is highly consistent with the theoretical stoichiometric ratio of the pure ODA-H molecule (C/N ≈ 18). These quantitative variations collectively verify the formation of a dense and robust ODA-H adsorption layer on the KCl surface, suggesting that the polar amino groups adsorb onto the mineral surface *via* electrostatic interaction (Table 3).

**Table 2:** Surface elemental composition (atomic %) of untreated KCl and ODA-H adsorbed KCl samples obtained from XPS analysis

Sample	C 1s	N 1s	K 2p	Cl 2p	C/N Ratio
Untreated KCl	16.44	0.71	26.10	25.12	23.15
After ODA-H adsorption	49.15	2.49	16.81	17.23	19.74

**Table 3:** High-resolution N 1s XPS peak-fitting parameters for untreated KCl and ODA-H adsorbed KCl

Sample	Component	Binding energy (eV)	FWHM (eV)	Area (%)
Untreated KCl	N 1s	399.30	3.37	0.71
After ODA-H adsorption	N 1s	399.48	3.37	2.49

The high-resolution N 1s spectrum exhibits a broad peak at 399.48 eV (FWHM = 3.37 eV), characteristic of neutral –NH_2_ groups. The exceptionally broad FWHM indicates a heterogeneous chemical environment, revealing a more complex adsorption mechanism than pure electrostatic interactions. This implies partial deprotonation of –NH_3_^+^ groups upon adsorption, forming neutral – NH_2_ species that participate in hydrogen bonding, while coexisting protonated species contribute to electrostatic attraction within the broad envelope. Notably, the unchanged K 2p and Cl 2p energies reflect the stability of the ionic KCl lattice and do not constitute conclusive evidence for a single adsorption mode. Overall, the XPS data point to a synergistic mechanism involving electrostatic attraction, partial deprotonation, and hydrogen bonding.

## 4 Conclusion

(1) ODA·HCl exhibits concentration-dependent self-assembly behavior in aqueous solution. At low concentrations (10^−5^–10^−4^ mol L^−1^), the grain size is approximately 2–3 µm. With increasing concentration, electrostatic repulsion between grains weakens, leading to reversible flocculation. The volume-average particle diameter (D[4,3]) increases from 7.44 µm to 30.1 µm, D50 rises from 6.17 µm to 21.4 µm, and the [(D90–D10)/D50] span increases significantly, indicating a decrease in the dispersion uniformity of the system.

(2) Within the actual flotation concentration range, the ODA·HCl suspension exhibit a main particle size distribution primarily between 4 and 256 µm. As the concentration increases, the median diameter (D50) shifts towards smaller sizes (*e.g.*, from 38.1 µm for P-1 to 25.6 µm for P-3), while the volume-average diameter (D[4,3]) fluctuates between 26.7 µm and 48.3 µm. Meanwhile, the relative span decreases to a lower range (from 2.68 for P-1 to 2.21 for P-6), indicating a trend of finer and more uniformly dispersed flocs at higher concentrations, despite the occasional presence of extremely large flocs (*e.g.*, D100 > 1000 µm). Regarding the acid anion effect, the sulfuric acid system causes intense agglomeration, the nitric acid system displays a bimodal distribution, the phosphoric acid system produces slightly coarser particles than the hydrochloric acid system, and the hydrochloric acid system yields the most optimal dispersion.

(3) Flotation recovery increases with ODA·HCl concentration (from 87.5% to 95.8%), while the required reagent volume decreases sharply (from 11.5 to 1.5 mL/100 g raw ore). At low concentrations, the larger volume of introduced water may potentially cause an estimated maximum KCl dissolution loss, whereas at high concentrations, excessive micelle formation during dynamic flotation reduces the effective adsorption efficiency. An intermediate concentration achieves an optimal balance among high recovery (>94%), favorable particle size distributions, and mother liquor equilibrium.

(4) FTIR and XPS confirm that ODA·HCl successfully adsorbs on the KCl surface. The characteristic absorption peaks (*γ*(NH_2_), *γ*(CH_2_), *δ*(NH_2_), *δ*(CH_2_)) and the N 1s spectrum (deconvoluted into protonated and partially deprotonated amine species) indicate a synergistic adsorption mechanism involving electrostatic attraction, partial deprotonation, and hydrogen bonding. The absence of chemical shifts in the K 2p and Cl 2p binding energies confirms that the ODA·HCl adsorption occurs on the surface without disrupting the KCl lattice.

This study reveals the behavioral of amine collectors from molecular micelles to flocs and ultimately to surface attachment, proposes a mechanism for the non-monotonic variation of adsorption efficiency under dynamic flotation, and provides new spectroscopic and kinetic evidence for the design of flotation reagent systems for salt minerals.

## Conflicts of interest

There are no conflicts to declare.

## Supplementary Material

RA-OLF-D6RA08381A-s001

## Data Availability

The authors confirm that the data supporting the findings of this study are available within the article. Supplementary information (SI): supplementary data associated with this article includes detailed particle size distribution (PSD) parameters and original distribution diagrams for ODA-H dispersions at different concentrations (Table S1 and Fig. S1–S11), as well as the detailed flotation experimental results (*n* = 3) with means and standard deviations for seven flotation test groups using different acidified ODA collectors (Tables S2–S8). See DOI: https://doi.org/10.1039/d6ra08381a.
