# Supplementary material for: Adsorption and precipitation of ODA on KCl surfaces during flotation: evidence from FTIR and XPS
Source: RSC Adv. 2026 Nov 2;16(49):67633–44. doi: 10.1039/d6ra08381a (PMC13725792; doi:10.1039/d6ra08381a)
Supplement: Supplementary file 1 [file RA-016-D6RA08381A-s001.pdf]

Supplementary Information (SI) for RSC Advances.
This journal is © The Royal Society of Chemistry

SUPPLEMENTARY INFORMATION

Adsorption and Precipitation of ODA on KCl Surfaces during Flotation: Evidence from FTIR and XPS

Huaide Cheng[a,b*], Changhao Luo[a,b,c], Qingyu Hai[a,b]

[a] *Key Laboratory of Green and High-end Utilization of Salt Lake Resources, Qinghai Institute of Salt Lakes, Chinese Academy of Sciences, Xining 810008, China*

[b] *Qinghai Provincial Key Laboratory of Geology and Environment of Salt Lakes, Xining 810008, China*

[c] *University of Chinese Academy of Sciences, Beijing, China*

*Corresponding author. Huaide Cheng, Qinghai Institute of Salt Lakes, Chinese Academy of Sciences, No 18 Xinning Road, Xi'ning, Qinghai 810008, China.

E-mail address: chenghuaide@isl.ac.cn (H. Cheng)

1. Particle size distribution (PSD) Data

Table S1 Summary of particle size distribution (PSD) parameters for ODA-H dispersions at different concentrations. *(All measurements were performed using a Malvern Mastersizer 3000+ with a wet dispersion unit; each sample was measured once, n = 1).*

Sample	D10 (μm)	D50 (μm)	D90 (μm)	D100 (μm)	D[4, 3] (μm)	Span =(D90-D10)/D50
1, 6.87×10^{-5} mol/L	2.01	6.17	14.9	27.4	7.44	2.09
2, 1.12×10^{-4} mol/L	2.24	7.32	18.6	31.1	9.02	2.23
3, 1.85×10^{-4} mol/L	3.39	14.7	48.1	110	20.9	3.04
4, 2.79×10^{-4} mol/L	3.30	13.3	42.7	97.8	18.7	2.96
5, 4.12×10^{-4} mol/L	4.53	21.4	69.5	163	30.1	3.04
P-1	10.6	38.1	113	1230	55.7	2.68
P-2	5.29	23.3	63.7	296	31.4	2.51
P-3	6.43	25.6	85.7	397	40.5	3.09
P-4	28.3	212	498	1390	250	2.21
P-5	3.56	12.9	73.0	184	26.7	5.38
P-6	12.2	35.9	91.6	504	48.3	2.21

The original particle size distribution diagrams of each sample are shown in Figures S1 to S11 respectively. Please refer to the following figures for details. In the figures, the horizontal axis represents the particle size classification (μm), and the vertical axis represents the volume density (%).

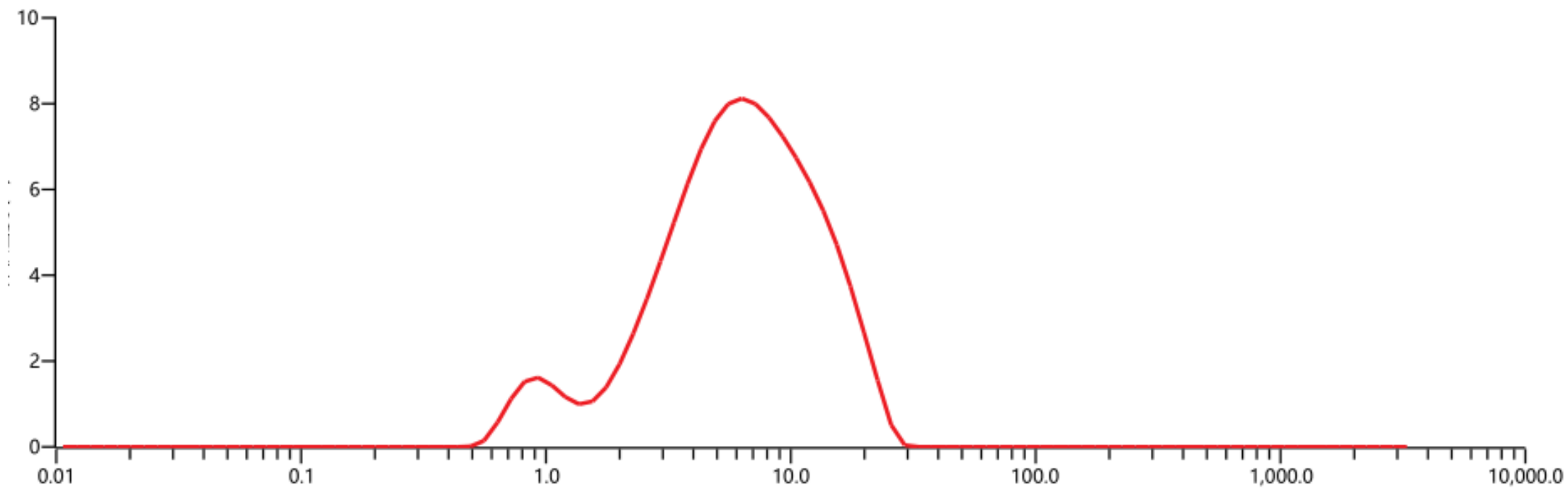

Figure S1 Sample 1, 6.87×10^{-5} mol/L

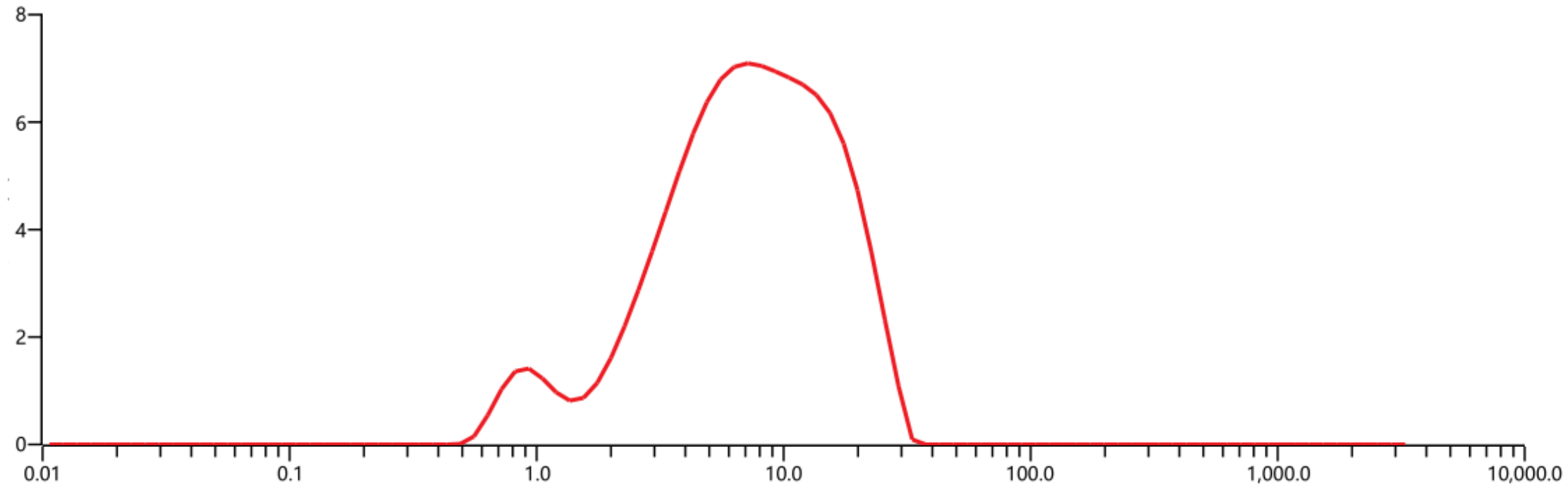

Figure S2 Sample 2, 1.12×10^{-4} mol/L

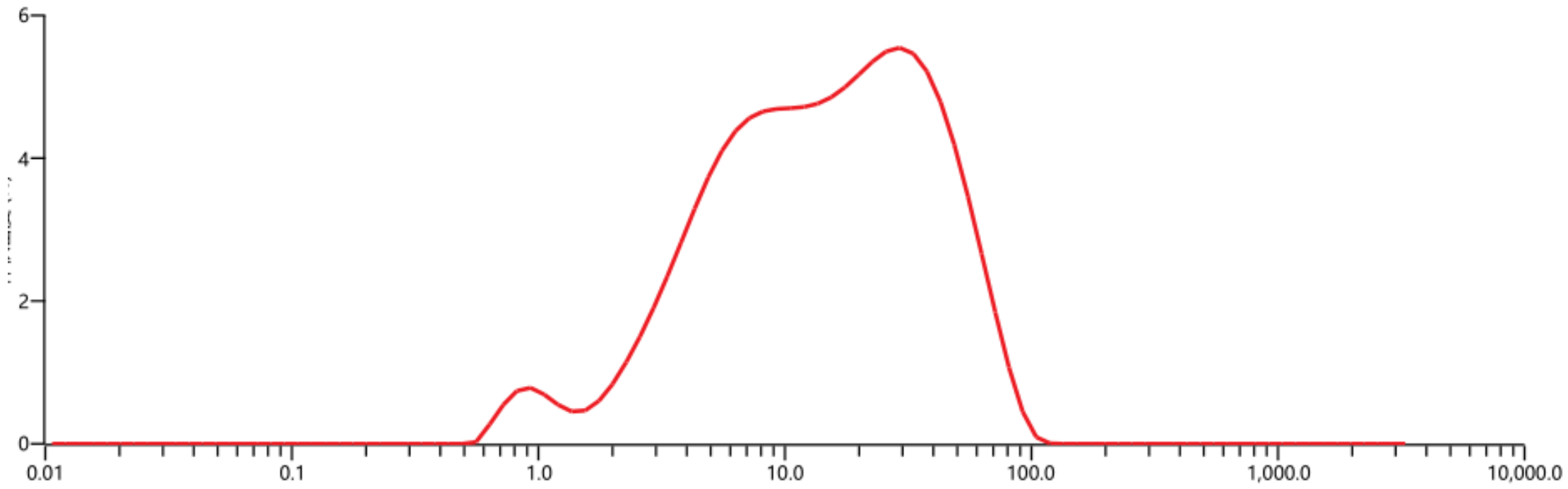

Figure S3 Sample 3, 1.85×10^{-4} mol/L

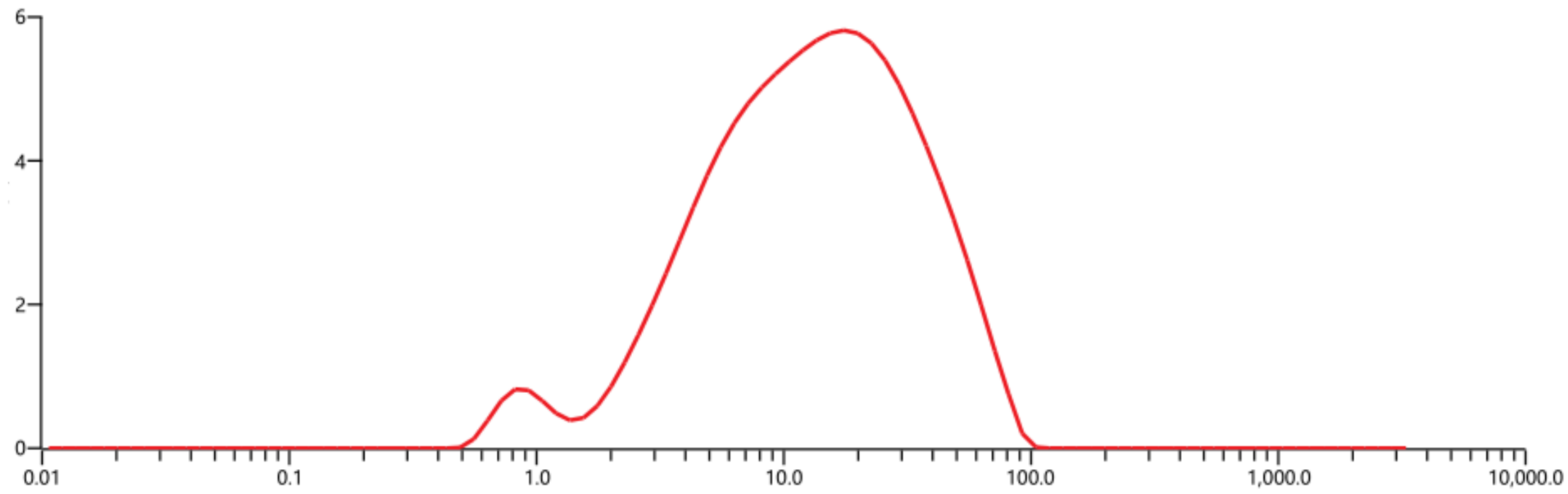

Figure S4 Sample 4, 2.79×10^{-4} mol/L

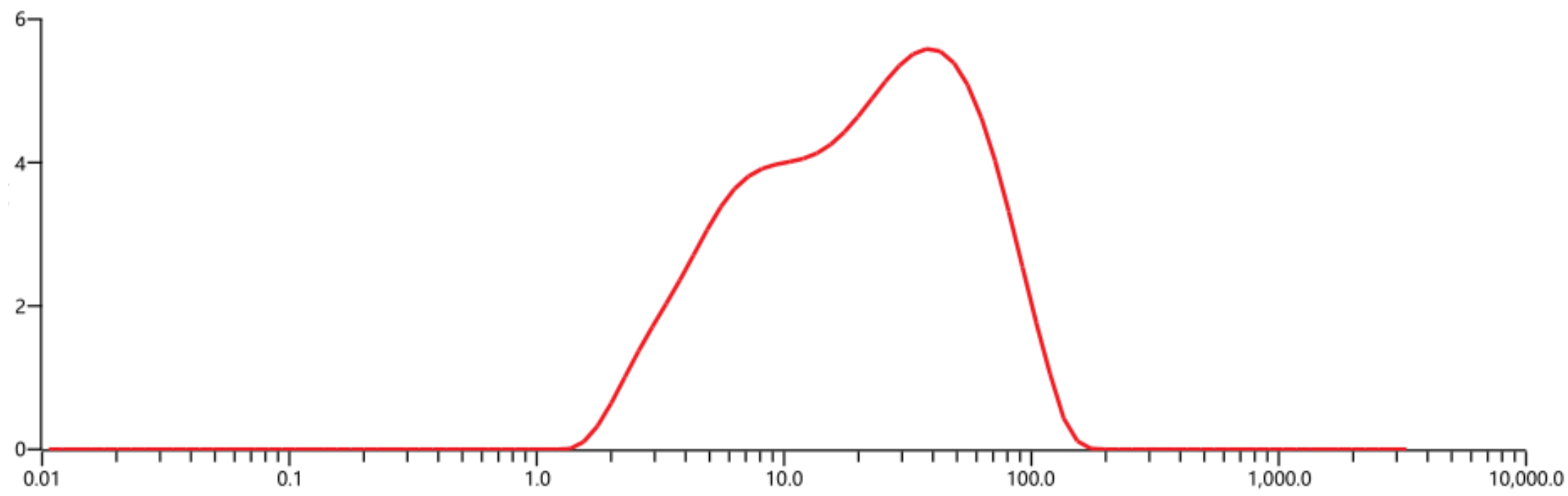

Figure S5 Sample 5, 4.12×10^{-4} mol/L

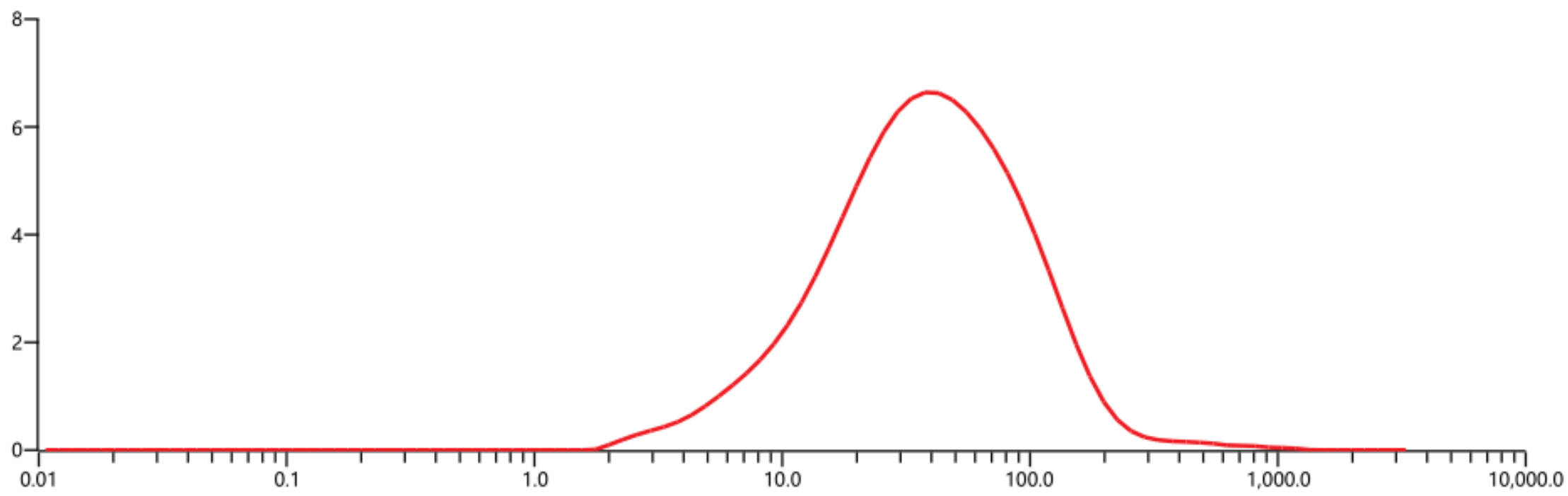

Figure S6 Sample P-1

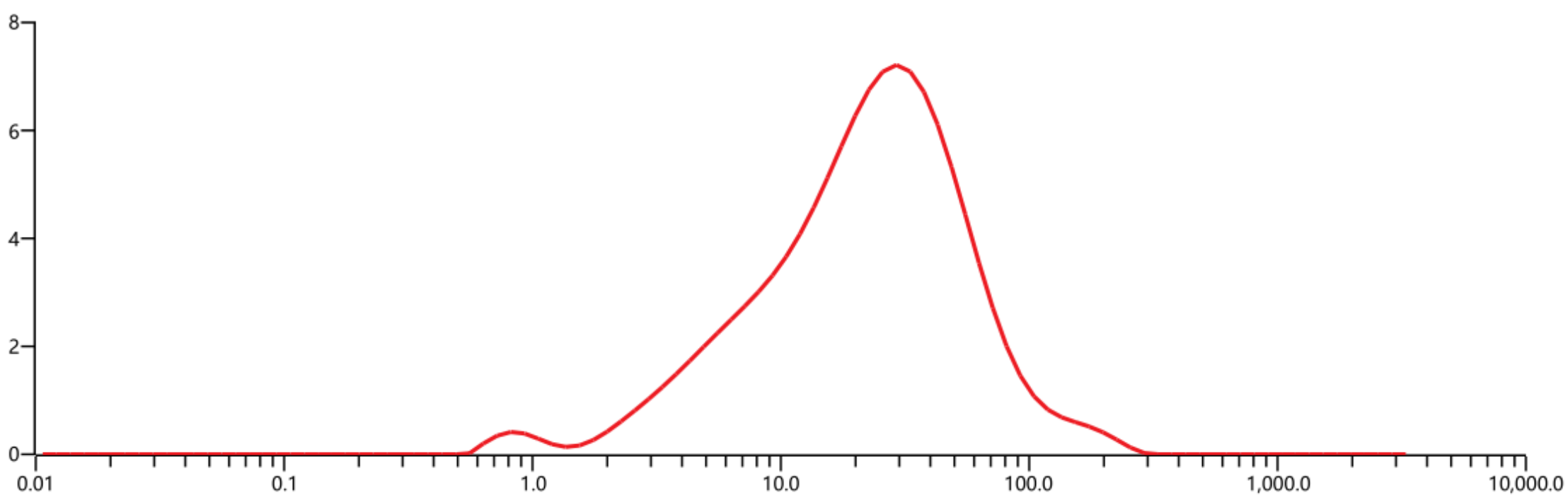

Figure S7 Sample P-2

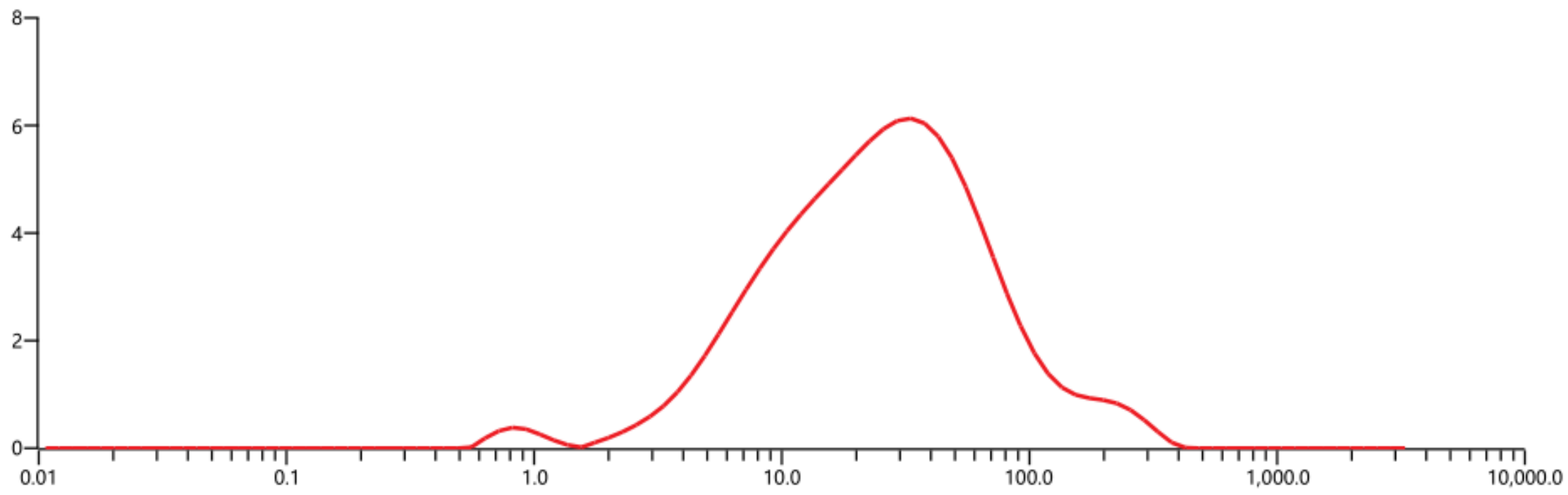

Figure S8 Sample P-3

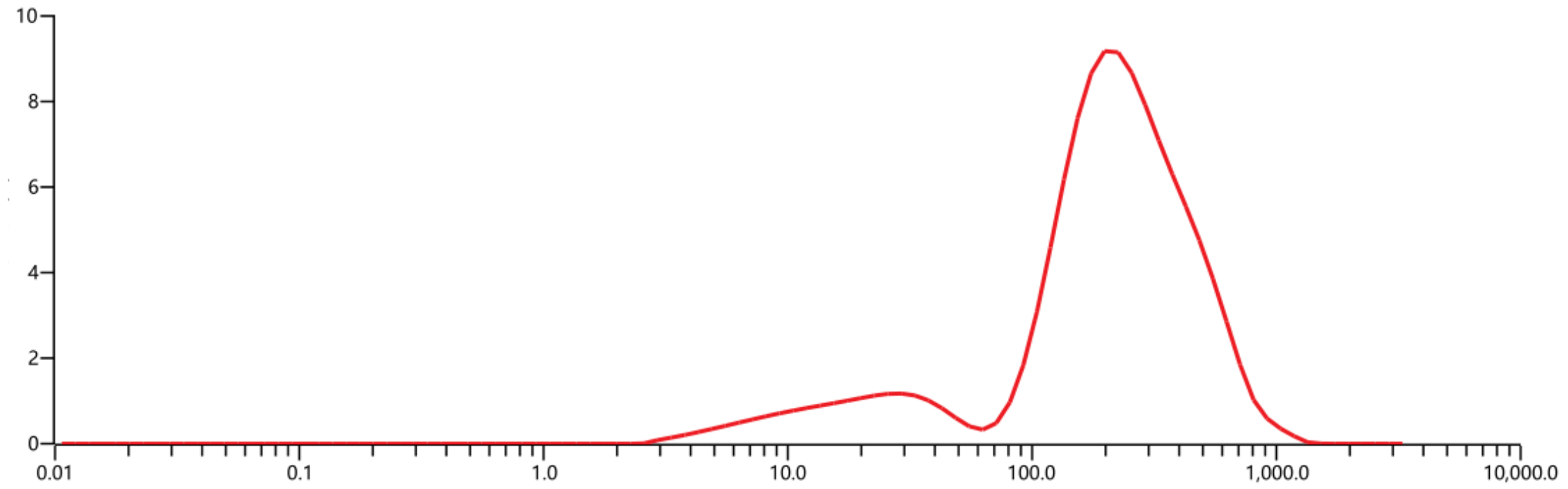

Figure S9 Sample P-4

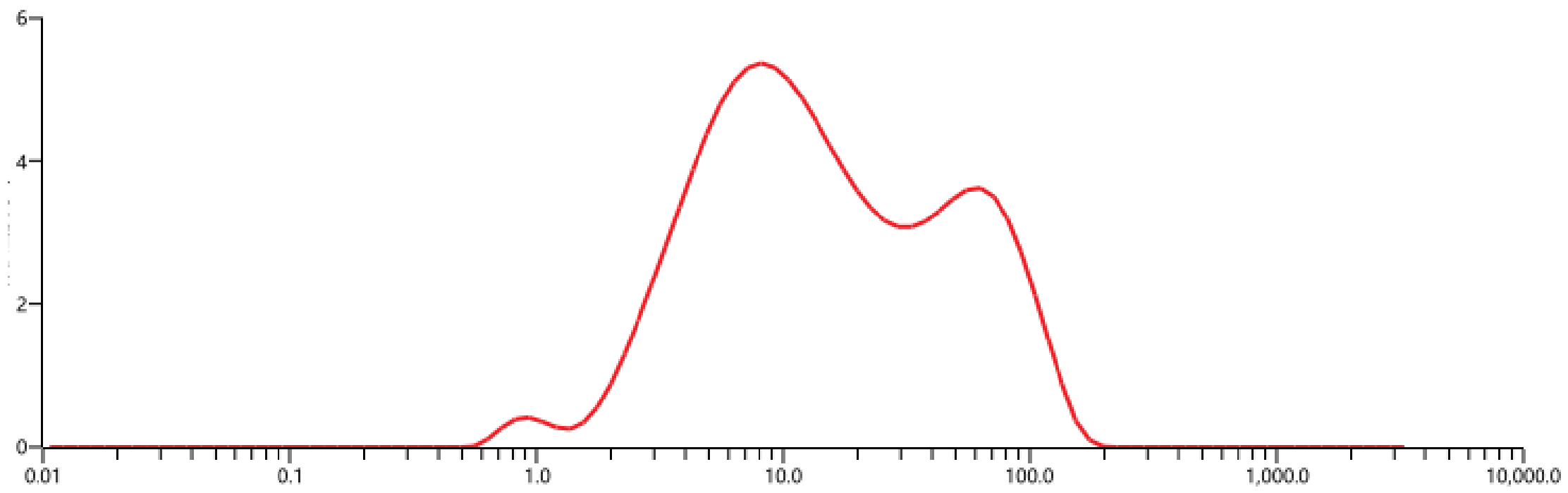

Figure S10 Sample P-5

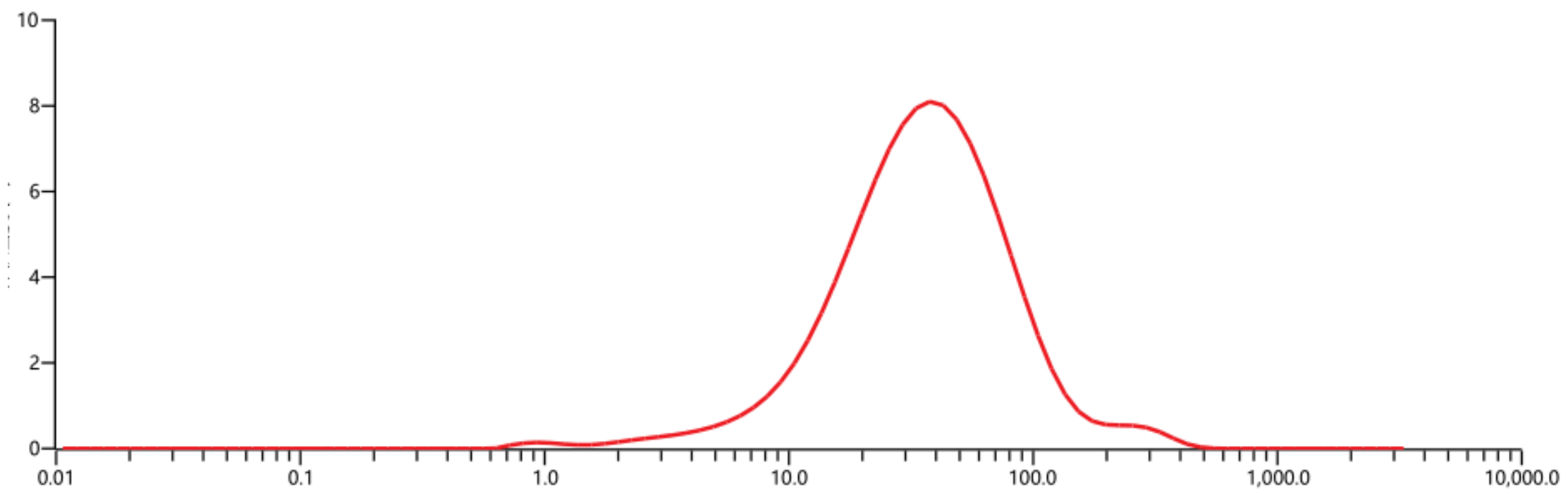

Figure S11 Sample P-6

2.Flotation Experiment

Flotation recovery was calculated as:

$$\text{Flotation recovery}(\%) = \frac{\text{Concentrate mass (g)}}{\text{Concentrate mass (g)} + \text{Tailings mass (g)}} \times 100\%$$

Table S2 Detailed flotation test results for Group 1 (n=3), ODA-HCl condition: concentration 5.23 $\times 10^{-3}$mol/L, dosage, 11.5mL

Group	Replicate	Feed mass (g)	Concentrate mass (g)	Tailings mass (g)	Flotation recovery (%)
1	1	100.00	87.56	13.26	86.85
	2	100.00	87.66	12.25	87.74
	3	100.00	88.23	11.96	88.06
Mean±SD					87.55±0.63
RSD (%)					0.72

Table S3 Detailed flotation test results for Group 2 (n=3), ODA-HCl condition: concentration 7.38×10^{-3}mol/L, dosage, 8.13mL

Group	Replicate	Feed mass (g)	Concentrate mass (g)	Tailings mass (g)	Flotation recovery (%)
2	1	100.00	91.66	8.17	91.82
	2	100.00	92.85	7.76	92.28
	3	100.00	91.05	8.95	91.05
Mean±SD					91.72±0.62
RSD (%)					0.68

Table S4 Detailed flotation test results for Group 3 (n=3), ODA-HCl condition: concentration 1.00×10^{-2}mol/L, dosage, 6mL

Group	Replicate	Feed mass (g)	Concentrate mass (g)	Tailings mass (g)	Flotation recovery (%)
3	1	100.00	94.46	5.86	94.16
	2	100.00	93.26	6.76	93.24
	3	100.00	93.88	6.86	93.19
Mean±SD					93.53±0.55
RSD (%)					0.58

Table S5 Detailed flotation test results for Group 4 (n=3), ODA-HCl condition: concentration 3.71×10^{-2}mol/L, dosage, 1.62mL

Group	Replicate	Feed mass (g)	Concentrate mass (g)	Tailings mass (g)	Flotation recovery (%)
4	1	100.00	100.43	4.37	95.83
	2	100.00	100.56	4.26	96.61
	3	100.00	100.47	4.42	95.23
Mean±SD					95.89±0.69
RSD (%)					0.72

Table S6 Detailed flotation test results for Group 5 (n=3), , ODA-H_2SO_4 condition: concentration 3.71 ×10^{-2}mol/L, dosage, 1.62mL

Group	Replicate	Feed mass (g)	Concentrate mass (g)	Tailings mass (g)	Flotation recovery (%)
5	1	100.00	44.98	51.54	46.60
	2	100.00	45.56	54.26	45.64
	3	100.00	46.36	50.85	47.69
Mean±SD					46.64±1.03
RSD (%)					2.20

Table S7 Detailed flotation test results for Group 6 (n=3), , ODA-HNO_3 condition: concentration 3.71 ×10^{-2}mol/L, dosage, 1.62mL

Group	Replicate	Feed mass (g)	Concentrate mass (g)	Tailings mass (g)	Flotation recovery (%)
6	1	100.00	90.28	7.83	92.02
	2	100.00	91.25	6.63	93.23
	3	100.00	90.55	8.63	91.30
Mean±SD					92.18±0.98
RSD (%)					1.06

Table S8 Detailed flotation test results for Group 7 (n=3), , ODA-H_3PO_4 condition: concentration 3.71 ×10^{-2}mol/L, dosage, 1.62mL

Group	Replicate	Feed mass (g)	Concentrate mass (g)	Tailings mass (g)	Flotation recovery (%)
7	1	100.00	83.55	13.01	86.53
	2	100.00	84.25	12.23	87.32
	3	100.00	82.56	14.02	85.48

Mean±SD		86.44±0.92
RSD (%)		1.07
